# Fluorescence resonance energy transfer in atomically precise metal nanoclusters by cocrystallization-induced spatial confinement

**DOI:** 10.1038/s41467-024-49735-7

**Published:** 2024-06-24

**Authors:** Hao Li, Tian Wang, Jiaojiao Han, Ying Xu, Xi Kang, Xiaosong Li, Manzhou Zhu

**Affiliations:** 1 Department of Chemistry and Centre for Atomic Engineering of Advanced Materials, Anhui University, 230601 Hefei, China; 2 Key Laboratory of Structure and Functional Regulation of Hybrid Materials of Ministry of Education, 230601 Hefei, China; 3 Key Laboratory of Functional Inorganic Material Chemistry of Anhui Province, Anhui University, 230601 Hefei, China; 4 School of Materials and Chemical Engineering, Anhui Jianzhu University, 230601 Hefei, China; 5 Department of Chemistry, University of Washington, Seattle, WA 98195-1653 USA

**Keywords:** Structural properties, Synthesis and processing

## Abstract

Understanding the fluorescence resonance energy transfer (FRET) of metal nanoparticles at the atomic level has long been a challenge due to the lack of accurate systems with definite distance and orientation of molecules. Here we present the realization of achieving FRET between two atomically precise copper nanoclusters through cocrystallization-induced spatial confinement. In this study, we demonstrate the establishment of FRET in a cocrystallized Cu_8_(*p*-MBT)_8_(PPh_3_)_4_@Cu_10_(*p*-MBT)_10_(PPh_3_)_4_ system by exploiting the overlapping spectra between the excitation of the Cu_10_(*p*-MBT)_10_(PPh_3_)_4_ cluster and the emission of the Cu_8_(*p*-MBT)_8_(PPh_3_)_4_ cluster, combined with accurate control over the confined space between the two nanoclusters. Density functional theory is employed to provide deeper insights into the role of the distance and dipole orientations of molecules to illustrate the FRET procedure between two cluster molecules at the electronic structure level.

## Abstract (web summary)

Understanding FRET of metal nanoparticles at the atomic level has long been a challenge. Here, the authors have achieved FRET activity with atomically precise Cu clusters by using a cocrystallisation-induced spatial confinement strategy.

## Introduction

Förster/fluorescence resonance energy transfer, a non-radiative energy transfer process, occurs through long-range dipole–dipole interactions between a donor–acceptor pair^1–3^. The term FRET is named after Theodor Förster, who proposed an equation to quantify the electronic excitation transfer efficiency from an energy donor to an acceptor, and the use of FRET as a spectroscopic or other technique has been in practice for over several decades^4^. Efficient FRET necessitates fulfilling the following conditions: (i) overlap between the emission spectrum of the donor and the excitation (or absorption) spectrum of the acceptor; (ii) small intermolecular distance between donor and acceptor; and (iii) favorable mutual orientation of their transition dipoles^5–7^. Over the past few decades, due to their ability to unravel fluorescence interactions between donor and acceptor with nanometer resolution, FRET-based sensors or imaging agents have found widespread applications in bio-related fields^8–10^. More recently, donor–acceptor composite materials have gained significant attention for their distance-dependent optoelectronic properties, which allow easy tuning of the energy transfer efficiency of the FRET system^11–14^. While FRET has been applied in various contexts, investigations into energy transfer efficiency have largely relied on semiempirical relationships^15–17^. Although the traditional FRET usually occurs based on atomically precise molecules, the relative position of molecules, the distance of molecules, and the orientation of transition dipoles were unclear in their solution systems, which hindered the directional design and modification of FRET materials.

An in-depth understanding of the energy transfer pathway at the quantum chemistry level remains challenging due to imprecise systems. In this context, the use of atomically precise systems with definite distance and orientation of molecules is a prerequisite for the deeply understanding of the FRET mechanism.

In the past few decades, nanoparticles have been developed as promising building blocks to construct FRET materials^18–22^. Atomically precise metal nanoclusters, a type of peculiar nanoparticles, have served as an emerging class of modular nanomaterials due to their advantage of programable geometric/electronic structures and physical/chemical properties^23–28^. Additionally, the development of metal clusters has progressed in exploring structure–property correlations at the atomic level due to their prominent quantum size effects and discrete electronic energy levels^29–34^. It has been demonstrated previously that nanoclusters can act as effective units to achieve efficient FRET^35–38^. However, for cluster-based intermolecular FRET systems, a clear perspective on the energy transfer mechanism remains inaccessible because of the imprecise structures or interactions between participating molecules^35,39,40^. Furthermore, although photoluminescence (PL) performance was exhibited in nanoclusters^41–44^, accomplishing the FRET between two discrete nanocluster systems remains challenging due to their potential instability and intercluster reaction activity^45–48^. Rationally developing an atomically precise cluster-based donor–acceptor system with FRET performance allows for an in-depth understanding of the intercluster energy transfer mechanism.

**Fig. 1:**
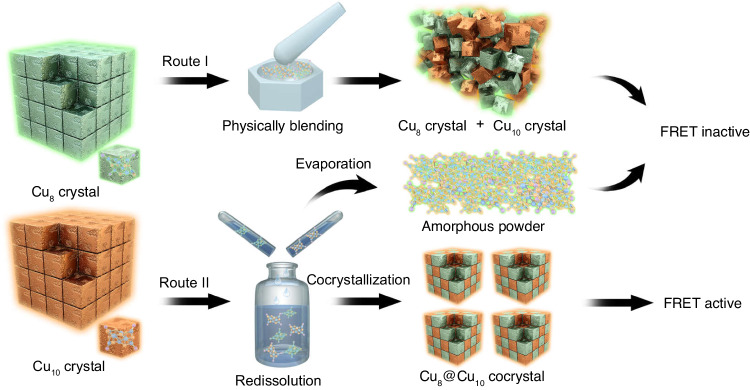
Illustration of the rational construction of FRET-active cluster systems. The requirements for FRET are: (i) overlapped excitation and emission and (ii) appropriate intermolecular distance. The route I represents the physically blended crystals of Cu_8_ and Cu_10_ clusters. The route II represents the forced cocrystallized Cu_8_@Cu_10_ cluster, which suits both requirements of FRET. Color labels: the crystals and the molecules in green represented the Cu_8_ cluster; the crystals and the molecules in orange represented the Cu_10_ cluster.

Herein, the FRET was achieved between nanoclusters at the atomic level by exploiting the cocrystallization-induced spatial confinement between two fluorescent copper clusters, Cu_8_(*p*-MBT)_8_(PPh_3_)_4_ (abbreviated as Cu_8_) and Cu_10_(*p*-MBT)_10_(PPh_3_)_4_ (abbreviated as Cu_10_), where *p*-MBT = 4-methylbenzenethiolate. We observed the partially overlapped spectra between the emission of Cu_8_ and the excitation of Cu_10_, demonstrating their potential for constructing a cluster-based FRET system. However, the physically blended crystals of Cu_8_ and Cu_10_ clusters were still FRET inactive due to the insufficiently small intermolecular distance (Fig. 1, route I). To address this, a spatial confinement strategy, i.e., the forced cocrystallization, was exploited between Cu_8_ and Cu_10_ clusters, leading to a cocrystallized bicomponent Cu_8_(*p*-MBT)_8_(PPh_3_)_4_@Cu_10_(*p*-MBT)_10_(PPh_3_)_4_ (abbreviated as Cu_8_@Cu_10_). The overlapped emission of the Cu_8_ donor and excitation of the Cu_10_ acceptor, along with their controllable intermolecular distance in the cocrystallized unit cell, endowed the Cu_8_@Cu_10_ cocrystal with the FRET characterization (Fig. 1, route II). Both experimental efforts and theoretical calculations were performed to illustrate the FRET mechanism by investigating the nonradiative energy transfer from the Cu_8_ donor to the Cu_10_ acceptor.

## Results

### Structure and PL performance

**Fig. 2:**
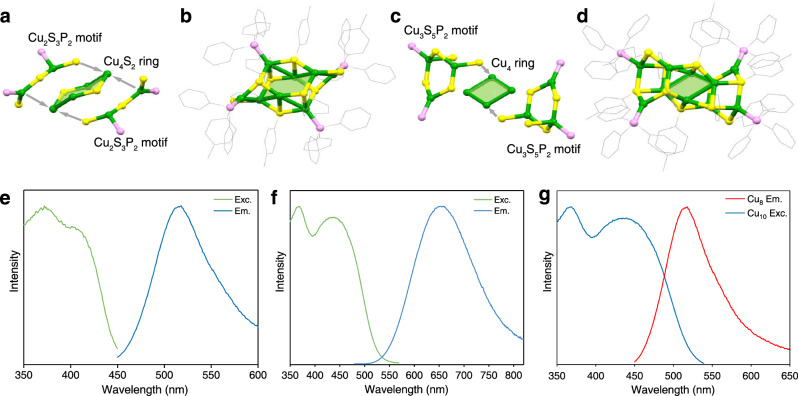
Structure and PL performance of Cu_8_ and Cu_10_ clusters. **a** Structural anatomy and **b** total structure of the Cu_8_ cluster. **c** Structural anatomy and **d** total structure of the Cu_10_ cluster. Color labels: green = Cu; yellow = S; pink = P; gray = C. All H atoms were omitted for clarity. The PL spectra of (**e**) Cu_8_ and **f** Cu_10_ clusters. Green lines: excitation spectra; blue lines: emission spectra. **g** Spectral overlap between the excitation spectrum of Cu_10_ (blue line) and the emission spectrum of Cu_8_ (red line).

The Cu_8_ and Cu_10_ clusters were obtained via a one-pot synthetic procedure, and their crystal structures were determined by single-crystal X-ray diffraction. Structurally, the Cu_8_ cluster was crystallized in a triclinic *P*−1 space group (Supplementary Fig. 1 and Supplementary Table 1), and its structure could be regarded as a chair conformation Cu_4_S_2_ hexatomic ring capped by two Cu_2_S_2_P_2_ motifs (Fig. 2a, b). The Cu–Cu distances ranged from 2.76 to 2.98 Å (Supplementary Table 2). The eight connective *p*-MBT ligands were bonded on the cluster surface by following two different coordination modes (*μ*_2_-S and *μ*_3_-S; Supplementary Fig. 2a). The Cu–P and Cu–S bond distances in Cu_8_ fell in the range of 2.23–2.24 and 2.23–2.41 Å, respectively (Supplementary Table 2). The Cu_10_ cluster was crystallized in a triclinic *P*−1 space group (Supplementary Fig. 3 and Supplementary Table 1), whose structure contained a rhombic Cu_4_ ring anchored by two Cu_3_S_5_P_2_ motifs at each end (Fig. 2c, d). The 10 *p*-MBT ligands also followed two different coordination modes on the Cu_10_ cluster surface (*μ*_2_-S and *μ*_3_-S; Supplementary Fig. 2b). The Cu–Cu bond lengths of Cu_10_ ranged from 2.74 to 2.99 Å. The Cu–P and Cu–S bond distances in Cu_10_ fell in the range of 2.23–2.24 and 2.23–2.41 Å, respectively (Supplementary Table 3). The compositions of Cu_8_ and Cu_10_ clusters were further verified by electrospray ionization mass spectrometry (Supplementary Fig. 4).

The photophysical properties of Cu_8_ and Cu_10_ clusters were then investigated. The Cu_8_ or Cu_10_ clusters were non-emissive in the solution state and displayed aggregation-induced emission (AIE) with the addition of a poor solvent, i.e., methanol (Supplementary Fig. 5)^49^. In contrast, Cu_8_ and Cu_10_ clusters in the crystal state exhibited significant PL at room temperature (Supplementary Fig. 6). Accordingly, all following optical properties of these clusters were tested in their crystal state. At room temperature, the Cu_8_ crystal displayed a maximum emission signal at 515 nm (*λ*_ex_ = 365 nm; Fig. 2e). The absolute PL quantum yield (QY) at room temperature of Cu_8_ was identified as 4.2% (Supplementary Fig. 7), and the average emission lifetime was 1.20 μs with three-lifetime components (*τ*_1_ = 4.12  μs, *τ*_2_ = 14.2 μs, and *τ*_3_ = 0.21 μs; Supplementary Fig. 8). The Cu_8_ cluster crystal showed enhanced PL intensity and red-shifted emission spectra in wavelength from 515 to 520 nm with decreasing temperature (Supplementary Fig. 9a, b). Although the PL changed significantly in the intensity of the Cu_8_ nanocluster (about 53.4 times, Supplementary Fig. 9c), the PL QY did not follow the same changing trend. Indeed, the QY value was also related to the absorption of the cluster sample. The absorption spectrum of Cu_8_ at 80 K was also measured to illustrate relative quantum yield at low temperatures. The results demonstrated that the absorption of Cu_8_ also displayed an enhancement in intensity with the temperature decreasing (about 2.47 times, Supplementary Fig. 9d). Thus, the relative quantum yield at 80 K of Cu_8_ nanocluster was given as 88.5%.

The crystalline state of Cu_10_ clusters showed strong orange emission (QY = 41.1%, Supplementary Fig. 10) with a maximum emission wavelength at 650 nm with a microsecond emission lifetime of 5.74 μs (*λ*_ex_ = 365 nm; Fig. 2f and Supplementary Fig. 11). The Cu_10_ cluster exhibited enhanced PL intensity and red-shifted emission from 650 to 690 nm with the decreased temperature (Supplementary Fig. 12). Both Cu nanoclusters were stable after the temperature-dependent PL test, which displayed a similar diffraction pattern confirmed by powder X-ray diffraction (PXRD; Supplementary Fig. 13). The shift of the maximum emission wavelength was due to the alternation of the PL emission mechanism at different temperatures^49,50^.

### Physical blending of crystals

We noticed that the excitation spectrum of the Cu_10_ cluster overlapped with the emission spectra of the Cu_8_ cluster at room temperature (Fig. 2g), suggesting the satisfaction of condition (i) for FRET between the Cu_8_ (as a donor) and Cu_10_ (as an acceptor) clusters. Therefore, we attempted to blend the single-component crystals of Cu_8_ and Cu_10_ clusters, and the mixture exhibited discrete PL of Cu_8_ and Cu_10_, regardless of the mole ratios between the two cluster compositions (Supplementary Fig. 14a). Due to the physical blending of Cu_8_ and Cu_10_ cluster crystals, the compositions of the mixture remained as crystals, and the molecule pair with effective FRET was not formed. In this context, the molecular distance between Cu_8_ and Cu_10_ cluster molecules was long and uncontrolled. To better control the molecular space of these two clusters, we blended the solution of Cu_8_ and Cu_10_ clusters and then made the solvent evaporation to obtain the amorphous powder solid mixture with closer intermolecular distances. The PL spectrum still displayed two-lifetime components corresponding to Cu_8_ and Cu_10_ clusters (Supplementary Fig. 14b). All these results above indicated that the PL of the Cu_8_ cluster was not quenched in the physical mixture sample of Cu_8_ and Cu_10_ clusters. In this condition, the construction of an effective intermolecular FRET system between two clusters was unsuccessful due to the uncontrolled distance and diploe orientation between Cu_8_ and Cu_10_.

For the construction of the FRET system between Cu_8_ and Cu_10_, the key factor is to confine the distance between the two cluster molecules, i.e., to accomplish the spatial confinement between them. In previous works, although the FRET process was achieved by using sliver nanoclusters such as Ag_16_ and Ag_29_^35,36^, the accurate dipole orientations, the favorable relative position for energy transfer, and the variation in the electronic structures of cluster molecules were hard to “see” directly in these cases. Besides, due to the various optical performances, for instance, the susceptible luminescence properties with different excitation source^51–53^, the adjustable emission wavelength^54,55^, and the multiple excited state^50,56^, copper-based nanoclusters have been exploited as potential candidates to accomplish the FRET. Recently, increasing research has focused on the cocrystallization of heterogeneous nanoclusters^57–61^. The correlated metal/ligand compositions of Cu_8_ and Cu_10_ clusters were expected to prevent potential metal/ligand exchange reactions and form a stable coexistence system.

### Achieving FRET through cocrystallisation

**Fig. 3:**
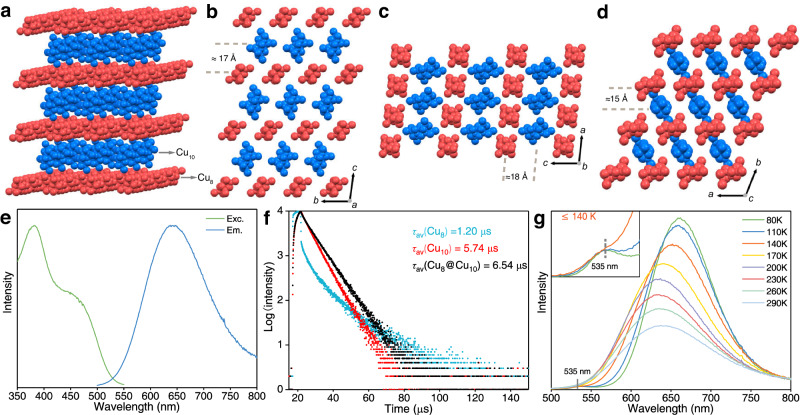
Structure and the PL performance of Cu_8_@Cu_10_ cocrystal. **a** The multilayer structure of cocrystallized Cu_8_ (color in red) and Cu_10_ (color in blue) clusters in the crystal lattice. **b–d** Packing of nanoclusters viewed from crystallographic *a*, *b*, and *c* axes, respectively, and the molecular distance between Cu_8_ and Cu_10_ clusters. **e** The PL spectra of Cu_8_@Cu_10_ cocrystal at room temperature. Green lines: excitation spectra; blue lines: emission spectra. **f** The emission lifetime of Cu_8_ (blue), Cu_10_ (red), and Cu_8_@Cu_10_ cocrystal (black) at room temperature. **g** Temperature-dependent PL spectra of cocrystallized Cu_8_ and Cu_10_ clusters (inset: the PL spectra below 140 K).

The forced cocrystallization was exploited between Cu_8_ and Cu_10_ clusters, giving rise to a cocrystallized bicomponent Cu_8_(*p*-MBT)_8_(PPh_3_)_4_@Cu_10_(*p*-MBT)_10_(PPh_3_)_4_. Before the crystallization, the ESI-MS of the mother liquid showed a mixed composition of Cu_8_ and Cu_10_ nanoclusters (Supplementary Fig. 15). The Cu_8_@Cu_10_ cluster crystallizes in a triclinic *P*−1 space group with a 1:1 molecular ratio (Fig. 3a, Supplementary Fig. 16 and Supplementary Table 1). The cocrystallized Cu_8_ and Cu_10_ molecules followed a layer-by-layer arrangement with intermolecular distances below 2 nm (i.e., the distance of molecular center; Fig. 3c, d). The Cu_8_@Cu_10_ crystal displayed a strong PL at 640 nm (QY = 43.3%; Fig. 3e and Supplementary Fig. 17) with a microsecond emission lifetime of 6.54 μs with two-lifetime components (*τ*_1_ = 1.72 μs and *τ*_2_ = 7.37 μs; Fig. 3f and Supplementary Fig. 18). The single emission peak at 640 nm of the cocrystallized Cu_8_@Cu_10_ clusters indicated that the fluorescence of the Cu_8_ cluster was quenched (Supplementary Fig. 19). The photophysical performance of cocrystallized Cu_8_@Cu_10_ was similar to that of Cu_10_, indicating that the FRET process was realized. In terms of the decay time, the Cu_8_@Cu_10_ crystal exhibited a longer lifetime (*τ*_av_ = 6.54 μs) than those of Cu_8_ (*τ*_av_ = 1.20 μs) and Cu_10_ (*τ*_av_ = 5.7 μs). The detailed photophysical data of Cu_8_, Cu_10_, and Cu_8_@Cu_10_ clusters at room temperature are listed in Supplementary Table 4. Temperature-dependent PL showed that the emission peak of the cocrystallized Cu_8_@Cu_10_ cluster at 640 nm was red-shifted to 660 nm with the temperature decreasing (Fig. 3g and Supplementary Fig. 20). The emission peak at 537 nm appeared when the temperature was below 140 K, and this peak was attributed to the Cu_8_ cluster (inset in Fig. 3g). The emergence of the 537 nm signal at low temperatures was rational. It might be due to the following two reasons: (i) the non-radiative process in the cocrystallization system was restricted, and (ii) the radiative transition (e.g., PL) of Cu_8_ was strengthened, which enhanced the PL QY sufficiently and the corresponding emission could be observed; indeed, the emerged 537 nm signal was similar to the emission of the monocomponent Cu_8_ nanocluster at low temperature^62,63^. The PXRD further confirmed that the crystal structure remained unchanged after the temperature-dependent PL test (Supplementary Fig. 21). Besides, the shifted PL wavelength of the Cu_8_@Cu_10_ cocrystal with its single components might result from the change of the electronic structure of clusters among the intermolecular assembly, which has been observed in previous works^44,64–66^. Collectively, the FRET was realized by confining the space among cluster molecules to fix the Cu_8_ and Cu_10_ clusters in a restricted space.

### Theoretical study of FRET process

Fermi’s golden rule:

1 $${k}_{{{{{{\rm{FRET}}}}}}}=\frac{2{{{{{\rm{\pi }}}}}}}{\hbar} {({V}_{{{{{{\rm{cp}}}}}}})}^{2}\, {{{{{\rm{FCWD}}}}}}$$

implied that the FRET rate is governed by the Franck–Condon factor weighted density of states (FCWD) realized by the spectra overlap and the electronic coupling strength (${V}_{{{{{{\rm{cp}}}}}}}$)^67^. Both key factors have been investigated by performing time-dependent density functional theory (TDDFT) calculations on Cu_8_, Cu_10_, and Cu_8_@Cu_10_ cocrystals, respectively.

**Fig. 4:**
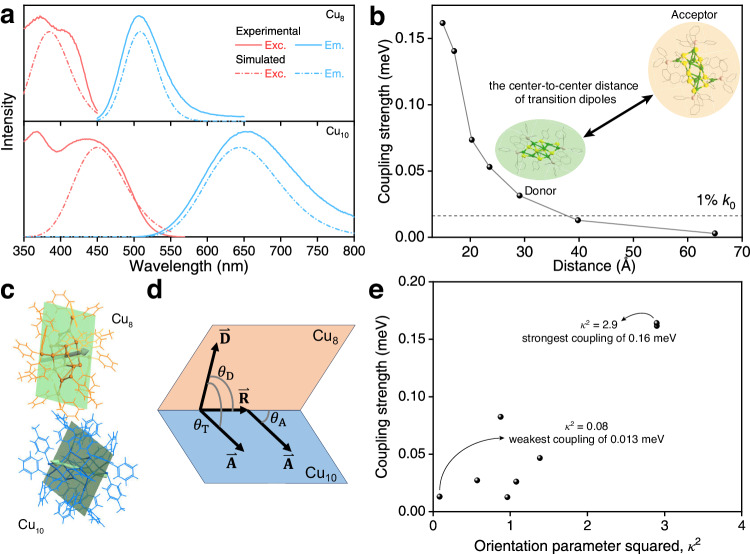
DFT calculations for the optical spectra and coupling strength of Cu_8_ and Cu_10_ clusters. **a** DFT calculated the absorption and emission spectra of Cu_8_ or Cu_10_ nanoclusters (solid lines). Dashed lines represented the experimental spectra. A uniform shift of 0.13 eV and adjusted Gaussian broadening were applied to the calculated spectra. Red lines: excitation spectra; blue lines: emission spectra. **b** The correlation between the electronic coupling strength and the center-to-center distance of transition dipoles. **c** Schematic diagram of the molecule plane for the Cu_8_@Cu_10_ cocrystal with green arrows labeling the best-fitted molecule plane. **d** Parameters for determining the orientation parameter (*κ*). **e** Calculated electronic coupling strength of transition dipoles with respect to the donor–acceptor orientation parameter squared (*κ*^2^). The orientation parameter (*κ*) and orientation parameter squared (*κ*^2^) are a unitless quantity.

As for the spectra overlap, the emission of Cu_8_ (2.31 eV) centered between the absorption (2.63 eV) and emission energy (1.80 eV) of Cu_10_ (Fig. 4a and Supplementary Fig. 22). The calculated absorption of Cu_10_ and emission of Cu_8_ displayed a 100 nm overlap from 450 to 550 nm, which satisfied the FRET requirement between the two clusters. The absorption and emission of Cu_8_ corresponded to the metal-to-ligand charge transfer (MLCT) from the Cu–S backbone to PPh_3_ ligands, and similar MLCT characterization was observed for the Cu_10_ nanocluster (Supplementary Fig. 23). In the Cu_8_@Cu_10_ cocrystal, as shown in Supplementary Fig. 24, the MLCT-corresponded excited state (S_1,A_) localized on the Cu_10_ cluster molecule (HOMO-1 to LUMO), while the excited state (S_1,D_) localized on the Cu_8_ cluster molecule (HOMO to LUMO + 1). The relative energies of the frontier orbitals of the Cu_8_@Cu_10_ cocrystal resembled a type-II alignment (Supplementary Fig. 25)^68–70^: the HOMO of Cu_8_ is higher than the HOMO of Cu_10_ and the LUMO of Cu_8_ is lower than the LUMO of Cu_10_. The larger gap on Cu_8_ satisfies the FRET from Cu_8_ to Cu_10_.

FRET is the energy transfer mechanism between donor and acceptor molecules. The donor (Cu_8_), initially pumped to its electronic excited state (S_1,D_), may transfer energy to excite the acceptor (Cu_10_) to its excited state (S_1,A_) through non-radiative coupling. The non-radiative coulombic interaction dipole-dipole between S_1,A_ and S_1,D_ corresponded to the FRET in the Cu_8_@Cu_10_ cocrystal. The coupling strength and estimated FRET rate are shown in Fig. 4b and Supplementary Table 5. In comparison, we investigated the direct radiative transition by evaluating the electric transition dipole moments 〈**i**|**−r**|**j**〉 and its oscillator strength (a unitless quantity, the detailed value sees Supplementary Table 6). The S_1,A_ → S_1,D_ oscillator strength was only 3.00 × 10^−5^. The low oscillator strength indicated a slow radiative transition rate. Therefore, the non-radiative FRET is the favored energy transfer mechanism between Cu_8_ and Cu_10_.

We also considered the possibility of Dexter energy transfer between Cu_8_ and Cu_10_ nanoclusters. Dexter energy transfer is the direct electron exchange process that requires the wavefunction overlap of HOMO (or LUMO) at the donor and acceptor, while the FRET rate is correlated with the transition dipole–dipole coupling strength (Supplementary Fig. 26). The spatial distribution of HOMO and LUMO at Cu_8_ (donor) and Cu_10_ (acceptor) is shown in Supplementary Fig. 27. The minimum distance between donor and acceptor LUMO (HOMO) is 10.7 (11.9) Å. Meanwhile, the overlap of donor and acceptor LUMO (HOMO) is negligible, indicating that the direct electron (hole) transfer is prohibited by the poor wavefunction overlap. Thus, the Dexter energy transfer is less favored in the Cu_8_@Cu_10_ cocrystal.

**Fig. 5:**
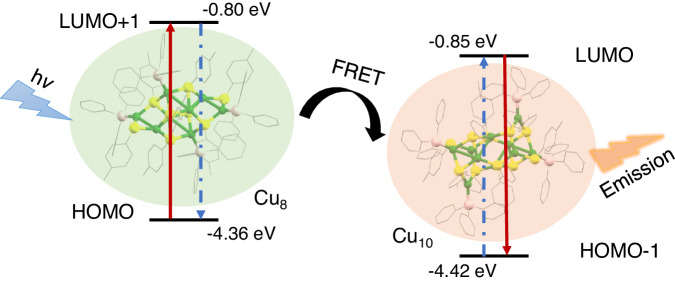
Energy diagram for the FRET process of the Cu_8_@Cu_10_ cocrystallized system. Color labels: green = Cu; yellow = S; pink = P; gray = C. All H atoms were omitted for clarity.

Collectively, the DFT calculations revealed the MLCT nature of the transition of Cu_8_ and Cu_10_ nanoclusters. Besides, the DFT calculations suggested that the FRET was induced by the energy transfer from S_1,D_ localized on Cu_8_ to S_1,A_ localized on Cu_10_ in the cocrystal, and a metal-to-ligand excitation on the Cu_8_ donor and a ligand-to-metal emission on the Cu_10_ acceptor was confirmed by the hole/electron spatial distribution (Supplementary Fig. 24). Therefore, the longer average PL lifetime of the cocrystallized Cu_8_@Cu_10_ than the monocomponent Cu_8_ or Cu_10_ might be attributed to it undergoing overall energy transfer processes including the excitation process of the Cu_8_ nanocluster, the FRET process, and the energy release process of the Cu_10_ nanocluster. Based on the above results, the brief energy transfer diagram for the FRET process of the Cu_8_@Cu_10_ cocrystallized system is given in Fig. 5.

In addition, we measured ${V}_{{{{{{\rm{cp}}}}}}}$ as the transition dipole–dipole interaction between S_1,A_ and S_1,D_ of the Cu_8_@Cu_10_ cocrystal. As shown in Fig. 4b and Supplementary Table 5, ${V}_{{{{{{\rm{cp}}}}}}}$ exhibited an obvious distance dependency. The center-to-center distance of the transition dipoles (Supplementary Fig. 28) of 1.49 nm in the cocrystal corresponded to a ${V}_{{{{{{\rm{cp}}}}}}}$ of 0.16 meV. The initial FRET rate *k*_0_ decayed to 1% when the distance was increased to 4 nm as *k*_FRET_ was proportional to ${{V}_{{{{{{\rm{cp}}}}}}}}^{2}$, which also accounted for the decreased FRET characterization in the non-crystalline phase.

Next, we tried to determine the Förster radius (*R*_0_). *R*_0_ satisfied the following equation:

2 $${R}_{0}=9.78 \times 10^{3}{({k}^{2}{Q}_{{{{{{\rm{D}}}}}}}{n}^{-4}{J}_{\lambda })}^{\frac{1}{6}}$$

where ${k}^{2}$ is the directional relationship of transition dipoles, ${Q}_{{{{{{\rm{D}}}}}}}$ is the quantum yield of the donor chromophore, *n* is the refractive index of the medium, and ${J}_{\lambda }$ is the spectral overlap of the donor and acceptor. In fact, the optical spectra of the two Cu nanoclusters were different between solution and crystalline phases, which might be attributed to the variation of their electronic structures in different states. These results could be inferred from the PL and UV–vis absorption spectra. As shown in Supplementary Fig. 29, the UV–vis absorption spectra of Cu_8_ and Cu_10_ nanoclusters in CH_2_Cl_2_ solution display no obvious absorption band (Supplementary Fig. 29a), while strong absorptions in the crystal state were detected (Supplementary Fig. 29b). In this context, we could not deduce the parameter of the refractive index of these cluster crystals from the solution state, and thus the Förster radius was incalculable. Furthermore, we calculated the FRET rate (*k*_FRET_) using the DFT-calculated coupling strength by exploiting Fermi’s golden rule. The FRET rate in Supplementary Table 5 is estimated by Fermi’s golden rule. In addition, the FRET parameters are further estimated given that:

3 $${k}_{{{{{{\rm{FRET}}}}}}}={k}_{{{{{{\rm{D}}}}}}}{\left(\frac{{R}_{0}}{r}\right)}^{6}$$

where ${k}_{{{{{{\rm{D}}}}}}}$ is the donor’s fluorescence decay rate in the absence of the acceptor and ${R}_{0}$ is the Förster radius. Then we can further relate ${V}_{{{{{{\rm{cp}}}}}}}$ and $r$ as

4 $${{V}_{{{{{{\rm{cp}}}}}}}}^{2}=\frac{{{\hbar }}{R}_{0}^{6}{k}_{{{{{{\rm{D}}}}}}}}{{{{{{\rm{FCWD}}}}}}2{{{{{\rm{\pi }}}}}}}{r}^{-6}$$

The linear fitting of ${{V}_{{{{{{\rm{cp}}}}}}}}^{2}$ versus ${r}^{-6}$ is shown in Supplementary Fig. 30. PL decay study has revealed ${k}_{{{{{{\rm{D}}}}}}}$ to be 2.01 × 10^6^ s^−1^. The FCWD was estimated to be 0.304 from the overlap of the normalized experimental spectra. Thus, the Förster radius ${R}_{0}$ was estimated to be 27.9 Å. Accordingly, the FRET efficiency (*E*_FRET_) in the different molecular distances was also given in Supplementary Table 5.

The favorable dipole orientations between the donor and the acceptor have been considered as another requirement to realize the FRET. Here, to verify the influence of dipole orientations of Cu_8_ cluster donors and Cu_10_ cluster acceptors on their FRET process, we redissolved the Cu_8_@Cu_10_ crystal after slight grinding due to the poor solubility of the crystal and then dropped the solution on the quartz plate for the solvent evaporation to form an amorphous powder. In this powder, the intermolecular distance and the dipole orientation of Cu_8_ and Cu_10_ nanoclusters were uncontrolled. Besides, the two copper nanoclusters might segregate into single separate crystal phases. In this context, the sample displayed a dual-emission spectrum corresponding to the emission of Cu_8_ and Cu_10_ clusters (Supplementary Fig. 31), indicating that the FRET was inactive in this amorphous powder.

To gain a deeper understanding of the dipole orientations between Cu_10_ and Cu_8_ and their effect on FRET, we investigated the energy transfer efficiency of each Cu_8_ @Cu_10_ donor-acceptor pair with different dipole orientations in the crystal lattice. The DFT calculated ${V}_{{{{{{\rm{cp}}}}}}}$ was employed to characterize the FRET rate between the Cu_8_ donor and the Cu_10_ acceptor with a fixed intermolecular distance but with different dipole–dipole orientations (Fig. 4c–e). The orientations were described by the orientation parameter squared (*κ*^2^, a unitless quantity) (Fig. 4c, d, and the detailed calculation method refers to Eq. (5) in the “Methods” section). In our simulation, *κ*^2^ ranged from 4 (dipoles are collinear) to 0 (dipoles are perpendicular). The strongest coupling (0.16 meV) was obtained for the near-collinear orientation (*κ*^2^ = 2.9). By comparison, the near-perpendicular orientation (*κ*^2^ = 0.08, corresponding to a ${V}_{{{{{{\rm{cp}}}}}}}$ of 0.013 meV) was less favored for FRET, resulting in a 150 times slower FRET rate (${{k}_{{{{{{\rm{FRET}}}}}}}\propto {V}_{{{\rm {cp}}}}}^{2}$). The DFT results demonstrated that the FRET rate could be significantly affected by the intermolecular orientation since the electronic coupling strength favored the collinear transition dipole-dipole orientation. As a result, it is rational that the disordered amorphous phase of the Cu_8_@Cu_10_ nanocluster exhibited a more inactive FRET characterization relative to its cocrystals.

## Discussion

In summary, we developed a spatial confinement system, i.e., the forced cocrystallized Cu_8_ and Cu_10_ clusters, for rationally realizing the FRET in atomically precise metal nanoclusters. In contrast to the FRET inactive cluster sample of the physically blended Cu_8_ and Cu_10_ in which only the overlap between the emission of the donor and the excitation of the acceptor was achieved, the cocrystallized Cu_8_@Cu_10_ sample confined the intramolecular spaces and favored the dipole orientations between cluster donor and acceptor, resulting in the realization of the FRET between cluster molecules. In addition to the experimental efforts, theoretical calculations were performed to verify the FRET between the Cu_8_ donor and the Cu_10_ acceptor in terms of the spectra overlap, the confined space, and the dipole orientation. Overall, the spatial confinement of the cocrystallized Cu_8_@Cu_10_ cluster system presented here is of significance because it provides an ideal platform to investigate the FRET mechanism in nanomaterials.

## Methods

### Reagents

All reagents are purchased from Sigma-Aldrich and used directly without further purification: cupric acetate monohydrate [(CH_3_COO)_2_Cu·H_2_O, 99.0%, metal basis], *p*-toluenethiol (C_7_H_7_S, *p*-MBT, 98%), triphenylphosphine (C_18_H_15_P, TPP, 99%), sodium borohydride (NaBH_4_, 99%), dichloromethane (CH_2_Cl_2_, HPLC grade), methanol (CH_3_OH, HPLC grade), *n*-hexane (C_6_H_14_, HPLC grade), and acetonitrile (CH_3_CN, HPLC grade).

### Synthesis of the Cu_8_(*p*-MBT)_8_(TPP)_4_ nanocluster

Copper acetate (0.25 mmol, 50 mg) was dissolved in 5 mL of acetonitrile, and then the solution was mixed in a round bottom flask containing 15 mL of dichloromethane. The solution was stirred vigorously at 1200 rpm. After 10 min, *p*-toluenethiol (45 mg, 0.37 mmol) was added, and the solution changed from blue-green to light yellow and turbid. After 60 min, triphenylphosphine (0.38 mmol, 100 mg) was added, and the solution gradually turned a light yellow and clarified. After 40 min, 3 mL of aqueous NaBH_4_ solution (0.53 mmol mL^−1^) was added. After 12 h, the aqueous phase was removed, and the organic phase was dried by rotary evaporation. The precipitate was dissolved with dichloromethane, and the solution was centrifuged to remove the byproducts. The yellow crystals of Cu_8_(*p*-MBT)_8_(TPP)_4_ were obtained by the liquid diffusion of *n*-hexane into the dichloromethane solution of the nanocluster for three days.

### Synthesis of the Cu_10_(*p*-MBT)_10_(TPP)_4_ nanocluster

Copper acetate (0.2 mmol, 40 mg) was dissolved in 5 mL of methanol, and then the solution was mixed in a round bottom flask containing 15 mL of dichloromethane. The solution was stirred vigorously at 1200 rpm. After 10 min, triphenylphosphine (0.19 mmol, 50 mg) was added. Then, after 30 min, *p*-toluenethiol (0.49 mmol, 60 mg) was added, and the color of the solution changed from blue-green to light yellow. After 4 h of the reaction, the organic solvent was evaporated to half by rotary evaporation, and then 5 mL of methanol was added. The mixed solution was evaporated at 4 °C, and then orange rod-shaped crystals were obtained.

### Synthesis of the Cu_8_(*p*-MBT)_8_(TPP)_4_@Cu_10_(*p*-MBT)_10_(TPP)_4_ cocrystallized nanocluster

The corresponding mother solutions were obtained according to the synthetic method of Cu_8_ and Cu_10_. The mother solutions of Cu_8_ and Cu_10_ clusters were mixed, and 5 mL of methanol was added to volatilize naturally for 24 h to obtain co-crystallized Cu_8_(*p*-MBT)_8_(TPP)_4_@Cu_10_(*p*-MBT)_10_(TPP)_4_.

### Single-crystal X-ray diffraction

The data collection for single-crystal X-ray diffraction of two Cu_7_ clusters was carried out on a Stoe Stadivari diffractometer under nitrogen flow using graphite-monochromatized Cu Kα radiation (*λ* = 1.54186 Å). Using Olex2^71^, the structure was solved with the ShelXT^72^ structure solution program using Intrinsic Phasing and refined with the ShelXL^73^ refinement package using least-squares minimization. All the non-hydrogen atoms were found directly. All the non-hydrogen atoms were refined anisotropically. All the hydrogen atoms were set in geometrically calculated positions and refined isotropically using a riding model. The diffuse electron densities resulting from the residual solvent molecules were removed from the data set using the SQUEEZE routine of PLATON and refined further using the data generated.

### Characterization

PL spectra, absolute PL quantum yield (PL QY), and emission lifetimes were measured on a HORIBA FluoroMax-4P. The absolute PL QY test was carried out by integrating the sphere at room temperature and calculated using the FluorEssence software. The PL lifetime was fitted by the DAS6 Analysis software. The PL lifetime of the Cu_8_ crystal was calculated by a third-order exponential fitting. The PL lifetime of Cu_10_ crystals was fitted by a first-order exponential fitting. The PL lifetime of Cu_8_@Cu_10_ crystals was fitted by a second-order exponential fitting. Electrospray ionization mass (ESI-MS) was performed on Waters XEVO G2-XS QTof mass spectrometer. The samples are dissolved in a mixture solution of CH_2_Cl_2_/CH_3_OH (v:v = 1:1), which is directly infused into the chamber at 10 µL min^−1^ with positive mode. X-ray diffraction (XRD) pattern was obtained on SmartLab 9KW with Cu Kα radiation. UV–vis absorption spectra in the solution state were collected on a PerkinElmer Lambda 465 spectrophotometer. UV–vis absorption spectra in the solid state were carried on a Shimadzu 3600-plus spectrophotometer with an integrating sphere.

### DFT calculations

All DFT calculations are performed using the Gaussian 16 package^74^. The PBE0 exchange correlation is adopted for all calculations^75^. The optimization for the ground state and the excited state employed hybrid basis sets: Def2-SVP for Cu, P, and S, and SBKJC-VDZ for C and H. As for the static calculations for the absorption and emission energies, the basis set is increased to Def2-SVP for all elements. The outer D and F basis are critical for describing the Cu–P bond. The excited states are analyzed by visualizing the spatial distribution of electrons and holes using Multiwfn^76,77^. The electron/hole isosurfaces are visualized by using VMD^78^. The absorption and emission spectra are plotted by applying a Gaussian broadening to the excitation with normalized oscillator strength. The broadening (ranging from 0.125 to 0.25 eV) is adjusted to match the experimental FWHM. The uniform broadened spectra with a narrow broadening of 0.125 eV are shown in Supplementary Fig. 22 to verify the spectra overlap and avoid manually introduced spectra overlap by over-broadening. A uniform shift of 0.13 eV is applied to the *x*-axis for all calculated spectra to be compared to the experimental spectra. The transition dipole–dipole coupling strength (${V}_{{{{{{\rm{cp}}}}}}}$) is estimated by employing the theoretical method as the formulations proposed by Iozzi, Mennucci, Tomasi, and Cammi, implemented in Gaussian 16^79^. To estimate the orientation parameter squared (*κ*^2^), the molecule plane is defined as shown in Fig. 4c. The orientation parameter (*κ*, a unitless quantity) is satisfied by the equation as follows^80^:

5 $${\kappa }^{2}={\left(\cos {\theta }_{{{{{{\rm{T}}}}}}}-3\cos {\theta }_{{{{{{\rm{D}}}}}}}\cos {\theta }_{{{{{{\rm{A}}}}}}}\right)}^{2}$$

where we define ${R}^{ \rightharpoonup }$ as the vector that is orthogonal to both the normal vector of the donor plane and acceptor plane. Thus, ${\theta }_{{{{{{\rm{D}}}}}}}$ is the angle between **R** and donor transition dipole moment. ${\theta }_{{{{{{\rm{A}}}}}}}$ is the angle between **R** and the acceptor transition dipole moment. ${\theta }_{{{{{{\rm{T}}}}}}}$ is the angle between the donor transition dipole and the acceptor transition dipole. The above parameters are shown in Fig. 4d.

### Supplementary information

Supplementary Information

Peer Review File

Description of Additional Supplementary Files

Supplementary Data 1

## Data Availability

The data that support the findings of this study are available from the corresponding authors upon request. Crystallographic data have been deposited at the Cambridge Crystallographic Data Centre (CCDC), under deposition numbers CCDC 2174160 (Cu_8_), 2174162 (Cu_10_), and 2174161 (Cu8@Cu_10_). Cartesian coordinates for the DFT calculations, as well as cif files, have been provided as a Supplementary Data file.

## References

[1] Sapsford KE, Berti L, Medintz IL (2006). Materials for fluorescence resonance energy transfer analysis: beyond traditional donor–acceptor combinations. Angew. Chem. Int. Ed..

[2] Scholes GD (2003). Long-range resonance energy transfer in molecular systems. Annu. Rev. Phys. Chem..

[3] Masters BR (2014). Paths to förster’s resonance energy transfer (FRET) theory. Eur. Phys. J. H.

[4] Förster T (1948). Zwischenmolekulare energiewanderung und fluoreszenz. Ann. Phys..

[5] Wu L (2020). Förster resonance energy transfer (FRET)-based small-molecule sensors and imaging agents. Chem. Soc. Rev..

[6] Algar WR, Hildebrandt N, Vogel SS, Medintz IL (2019). FRET as a biomolecular research tool—understanding its potential while avoiding pitfalls. Nat. Methods.

[7] Pramanik A (2020). Forster resonance energy transfer assisted white light generation and luminescence tuning in a colloidal graphene quantum dot-dye system. J. Colloid Interface Sci..

[8] Liang Z (2022). Ratiometric FRET Encoded Hierarchical ZrMOF @ Au cluster for ultrasensitive quantifying MicroRNA in vivo. Adv. Mater..

[9] Giepmans BNG, Adams SR, Ellisman MH, Tsien RY (2006). The fluorescent toolbox for assessing protein location and function. Science.

[10] Agam G (2023). Reliability and accuracy of single-molecule FRET studies for characterization of structural dynamics and distances in proteins. Nat. Methods.

[11] Tummeltshammer C (2017). On the ability of Förster resonance energy transfer to enhance luminescent solar concentrator efficiency. Nano Energy.

[12] Coffey DC, Ferguson AJ, Kopidakis N, Rumbles G (2010). Photovoltaic charge generation in organic semiconductors based on long-range energy transfer. ACS Nano.

[13] Zhang B, Lyu G, Kelly EA, Evans RC (2022). Förster resonance energy transfer in luminescent solar concentrators. Adv. Sci..

[14] Daiber B, van den Hoven K, Futscher MH, Ehrler B (2021). Realistic efficiency limits for singlet-fission silicon solar cells. ACS Energy Lett..

[15] Huang C-S (2022). Amphiphilic polymer co-network: a versatile matrix for tailoring the photonic energy transfer in wearable energy harvesting devices. Adv. Energy Mater..

[16] Park Y (2008). Hybrid CdSe nanoparticle-carbazole dendron boxes: electropolymerization and energy-transfer mechanism shift. Adv. Funct. Mater..

[17] Zhou B, Yan D (2019). Hydrogen-bonded two-component ionic crystals showing enhanced long-lived room-temperature phosphorescence via TADF-assisted Förster resonance energy transfer. Adv. Funct. Mater..

[18] Siefe C (2019). Sub-20 nm core–shell–shell nanoparticles for bright upconversion and enhanced Förster resonant energy transfer. J. Am. Chem. Soc..

[19] Lessard-Viger M, Rioux M, Rainville L, Boudreau D (2009). FRET enhancement in multilayer core-shell nanoparticles. Nano Lett..

[20] Yao J, Yang M, Duan Y (2014). Chemistry, biology, and medicine of fluorescent nanomaterials and related systems: new insights into biosensing, bioimaging, genomics, diagnostics, and therapy. Chem. Rev..

[21] Sarkar S, Bose R, Jana S, Jana NR, Pradhan N (2010). Doped semiconductor nanocrystals and organic dyes: an efficient and greener FRET system. J. Phys. Chem. Lett..

[22] Bain D, Maity S, Patra A (2019). Opportunities and challenges in energy and electron transfer of nanocluster based hybrid materials and their sensing applications. Phys. Chem. Chem. Phys..

[23] Yao Q (2023). Supercrystal engineering of atomically precise gold nanoparticles promoted by surface dynamics. Nat. Chem..

[24] Desireddy A (2013). Ultrastable silver nanoparticles. Nature.

[25] Li Y (2021). Double-helical assembly of heterodimeric nanoclusters into supercrystals. Nature.

[26] Jadzinsky PD, Calero G, Ackerson CJ, Bushnell DA, Kornberg RD (2007). Structure of a thiol monolayer-protected gold nanoparticle at 1.1 Å resolution. Science.

[27] Wang Z-Y (2018). Atomically precise site-specific tailoring and directional assembly of superatomic silver nanoclusters. J. Am. Chem. Soc..

[28] De Nardi M (2014). Gold nanowired: a linear (Au_25_)_*n*_ polymer from Au_25_ molecular clusters. ACS Nano.

[29] Jin R (2010). Quantum sized, thiolate-protected gold nanoclusters. Nanoscale.

[30] Soldan G (2016). Gold doping of silver nanoclusters: a 26-fold enhancement in the luminescence quantum yield. Angew. Chem. Int. Ed..

[31] Zhu C (2022). Fluorescence or phosphorescence? The metallic composition of the nanocluster kernel does matter. Angew. Chem. Int. Ed..

[32] Nguyen T-AD (2015). A Cu_25_ nanocluster with partial Cu(0) character. J. Am. Chem. Soc..

[33] Yang H (2017). Embryonic growth of face-center-cubic silver nanoclusters shaped in nearly perfect half-cubes and cubes. J. Am. Chem. Soc..

[34] Yu Y (2014). Identification of a highly luminescent Au_22_(SG)_18_ nanocluster. J. Am. Chem. Soc..

[35] Das AK, Biswas S, Manna SS, Pathak B, Mandal S (2022). An atomically precise silver nanocluster for artificial light-harvesting system through supramolecular functionalization. Chem. Sci..

[36] Pyo K (2023). Atomistic view of the energy transfer in a fluorophore-functionalized gold nanocluster. J. Am. Chem. Soc..

[37] Mondal K (2022). Self-assembly of solvent-stabilized au nanocluster as efficient Förster resonance energy-transfer initiator for white light generation. J. Phys. Chem. Lett..

[38] Xiao Y, Wu Z, Yao Q, Xie J (2021). Luminescent metal nanoclusters: biosensing strategies and bioimaging applications. Aggregate.

[39] Biswas S (2023). Luminescent [CO_2_@Ag_20_(SAdm)_10_(CF_3_COO)_10_(DMA)_2_] nanocluster: synthetic strategy and its implication towards white light emission. Nanoscale.

[40] Packirisamy V, Pandurangan P (2022). Interaction of atomically precise thiolated copper nanoclusters with proteins: a comparative study. ACS Omega.

[41] Kang X, Zhu M (2019). Tailoring the photoluminescence of atomically precise nanoclusters. Chem. Soc. Rev..

[42] Huang R-W (2017). Hypersensitive dual-function luminescence switching of a silver-chalcogenolate cluster-based metal-organic framework. Nat. Chem..

[43] Kang X (2019). Rational construction of a library of M_29_ nanoclusters from monometallic to tetrametallic. Proc. Natl Acad. Sci. USA.

[44] Sugiuchi M (2017). Aggregation-induced fluorescence-to-phosphorescence switching of molecular gold clusters. J. Am. Chem. Soc..

[45] Chakraborty P (2019). Rapid isotopic exchange in nanoparticles. Sci. Adv..

[46] Chakraborty P (2023). Elucidating the structures of intermediate fragments during stepwise dissociation of monolayer-protected silver clusters. Angew. Chem. Int. Ed.

[47] Neumaier M (2021). Kinetics of intercluster reactions between atomically precise noble metal clusters [Ag_25_(DMBT)_18_]^−^ and [Au_25_(PET)_18_]^−^ in room temperature solutions. J. Am. Chem. Soc..

[48] Tang L (2021). Dynamic metal exchange between a metalloid silver cluster and silver(I) thiolate. Inorg. Chem..

[49] Sun P-P (2022). Real-time fluorescent monitoring of kinetically controlled supramolecular self-assembly of atom-precise Cu_8_ nanocluster. Angew. Chem. Int. Ed..

[50] Li B, Fan H-T, Zang S-Q, Li H-Y, Wang L-Y (2018). Metal-containing crystalline luminescent thermochromic materials. Coord. Chem. Rev..

[51] Yuan P (2023). Hybrid thermally activated nanocluster fluorophores for X-ray scintillators. ACS Energy Lett..

[52] Huang R-W (2023). Radioluminescent Cu–Au metal nanoclusters: synthesis and self-assembly for efficient X-ray scintillation and imaging. J. Am. Chem. Soc..

[53] Xu C (2023). A High-nuclearity copper sulfide nanocluster [S-Cu_50_] featuring a double-shell structure configuration with Cu(II)/Cu(I) valences. J. Am. Chem. Soc..

[54] Peng S-K, Yang H, Luo D, Ning G-H, Li D (2024). A highly NIR emissive Cu_16_Pd_1_ nanocluster. Small.

[55] Ma X-H (2023). High-efficiency pure blue circularly polarized phosphorescence from chiral N-heterocyclic-carbene-stabilized copper(I) clusters. J. Am. Chem. Soc..

[56] Shi Y-e, Ma J, Feng A, Wang Z, Rogach AL (2021). Aggregation-induced emission of copper nanoclusters. Aggregate.

[57] Huang J-H, Liu L-Y, Wang Z-Y, Zang S-Q, Mak TCW (2022). Modular cocrystallization of customized carboranylthiolate-protected copper nanoclusters via host–guest interactions. ACS Nano.

[58] He L, Gan Z, Xia N, Liao L, Wu Z (2019). Alternating array stacking of Ag_26_Au and Ag_24_Au nanoclusters. Angew. Chem. Int. Ed..

[59] Liu J-Y (2019). Different silver nanoparticles in one crystal: Ag_210_(^*i*^PrPhS)_71_(Ph_3_P)_5_Cl and Ag_211_(^*i*^PrPhS)_71_(Ph_3_P)_6_Cl. Angew. Chem. Int. Ed..

[60] Yan J (2018). Co-crystallization of atomically precise metal nanoparticles driven by magic atomic and electronic shells. Nat. Commun..

[61] Liu D (2021). Interdependence between nanoclusters AuAg_24_ and Au_2_Ag_41_. Nat. Commun..

[62] Kang X, Wang S, Zhu M (2018). Observation of a new type of aggregation-induced emission in nanoclusters. Chem. Sci..

[63] Han Z (2020). Ultrastable atomically precise chiral silver clusters with more than 95% quantum efficiency. Sci. Adv..

[64] Wu Z (2019). Aurophilic interactions in the self-assembly of gold nanoclusters into nanoribbons with enhanced luminescence. Angew. Chem. Int. Ed..

[65] Li H (2022). Triple-helical self-assembly of atomically precise nanoclusters. J. Am. Chem. Soc..

[66] Nag A (2018). Polymorphism of Ag_29_(BDT)_12_(TPP)_4_^3−^ cluster: interactions of secondary ligands and their effect on solid state luminescence. Nanoscale.

[67] Yin J, Brédas J-L, Bakr OM, Mohammed OF (2020). Boosting self-trapped emissions in zero-dimensional perovskite heterostructures. Chem. Mater..

[68] Ihn, T. *Semiconductor Nanostructures: Quantum States and Electronic Transport* (Oxford University Press, 2009).

[69] Zhang J, Zhang M, Sun R-Q, Wang X (2012). A facile band alignment of polymeric carbon nitride semiconductors to construct isotype heterojunctions. Angew. Chem. Int. Ed..

[70] Hu W, Yang J (2017). Two-dimensional van der Waals heterojunctions for functional materials and devices. J. Mater. Chem. C.

[71] Dolomanov OV (2009). OLEX2: a complete structure solution, refinement and analysis program. J. Appl. Crystallogr..

[72] Sheldrick GM (2015). SHELXT-integrated space-group and crystal-structure determination. Acta Crystallogr..

[73] Sheldrick GM (2015). Crystal structure refinement with SHELXL. Acta Crystallogr..

[74] Frisch, M. J. et al. *Gaussian 16, Revision B.01* (Gaussian, Inc., Wallingford, CT, 2016).

[75] Adamo C, Barone V (1999). Toward reliable density functional methods without adjustable parameters: the PBE0 model. J. Chem. Phys..

[76] Lu T, Chen F (2012). Multiwfn: a multifunctional wavefunction analyzer. J. Comput. Chem..

[77] Liu Z, Lu T, Chen Q (2020). An *sp*-hybridized all-carboatomic ring, cyclo[18]carbon: bonding character, electron delocalization, and aromaticity. Carbon.

[78] Humphrey W, Dalke A, Schulten K (1996). VMD: visual molecular dynamics. J. Mol. Graph..

[79] Iozzi MF, Mennucci B, Tomasi J, Cammi R (2004). Excitation energy transfer (EET) between molecules in condensed matter: a novel application of the polarizable continuum model (PCM). J. Chem. Phys..

[80] van der Meer BW (2002). Kappa-squared: from nuisance to new sense. Rev. Mol. Biotechnol..

