# Supplementary material for: Fluorescence resonance energy transfer in atomically precise metal nanoclusters by cocrystallization-induced spatial confinement
Source: Nat Commun. 2024 Jun 24;15:5351. doi: 10.1038/s41467-024-49735-7 (PMC11196639; doi:10.1038/s41467-024-49735-7)
Supplement: Supplementary file 1 — Supplementary Information [file 41467_2024_49735_MOESM1_ESM.pdf]

Supplementary Information

Fluorescence resonance energy transfer in atomically precise metal nanoclusters by cocrystallization-induced spatial confinement

Hao Li[1,2,3,4,#], Tian Wang[5,#], Jiaojiao Han[1,2,3,#], Ying Xu[1,2,3], Xi Kang[1,2,3,✉], Xiaosong Li[4,✉], Manzhou Zhu[1,2,3,✉]

[1]Department of Chemistry and Centre for Atomic Engineering of Advanced Materials, Anhui University, Hefei, 230601, China.

[2]Key Laboratory of Structure and Functional Regulation of Hybrid Materials of Ministry of Education, Hefei, 230601, China.

[3]Key Laboratory of Functional Inorganic Material Chemistry of Anhui Province, Anhui University, Hefei, 230601, China.

[4]School of Materials and Chemical Engineering, Anhui Jianzhu University, Hefei, 230601, China.

[5]Department of Chemistry, University of Washington, Seattle, WA 98195-1653, United States.

[#]These authors contributed equally.

[✉]Email:

kangxi_chem@ahu.edu.cn (X.K.)

xsli@uw.edu (X.L.)

zmz@ahu.edu.cn (M.Z.)

Table of contents **Pages**

Supplementary Figures 1-31	1-12
Supplementary Tables 1-6	13-15
Supplementary Notes 1-3	15-20
Supplementary References	20

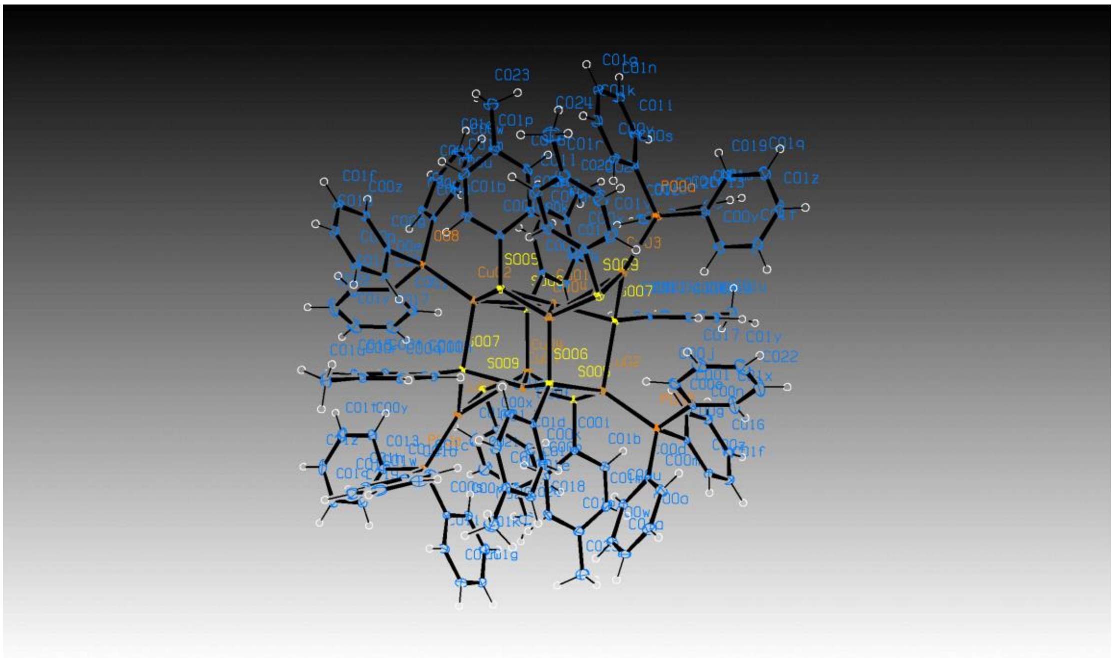

Supplementary Fig. 1. Representative Ortep diagram of Cu_8.[1] Thermal ellipsoids are set at 30% probability level. The CCDC number is 2174160.

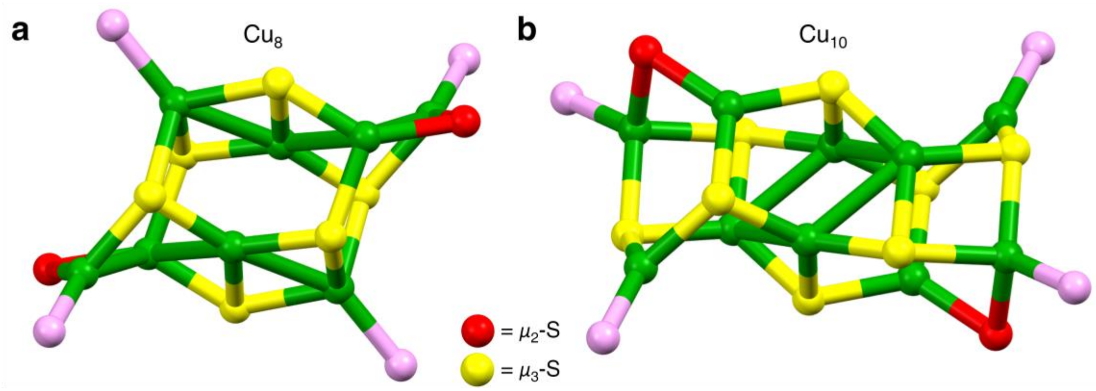

Supplementary Fig. 2. Multifarious coordination modes of S atoms in (**a**) Cu_8 and (**b**) Cu_{10} clusters. Color labels: green = Cu; yellow/red = S; pink = P. All C and H atoms were omitted for clarity.

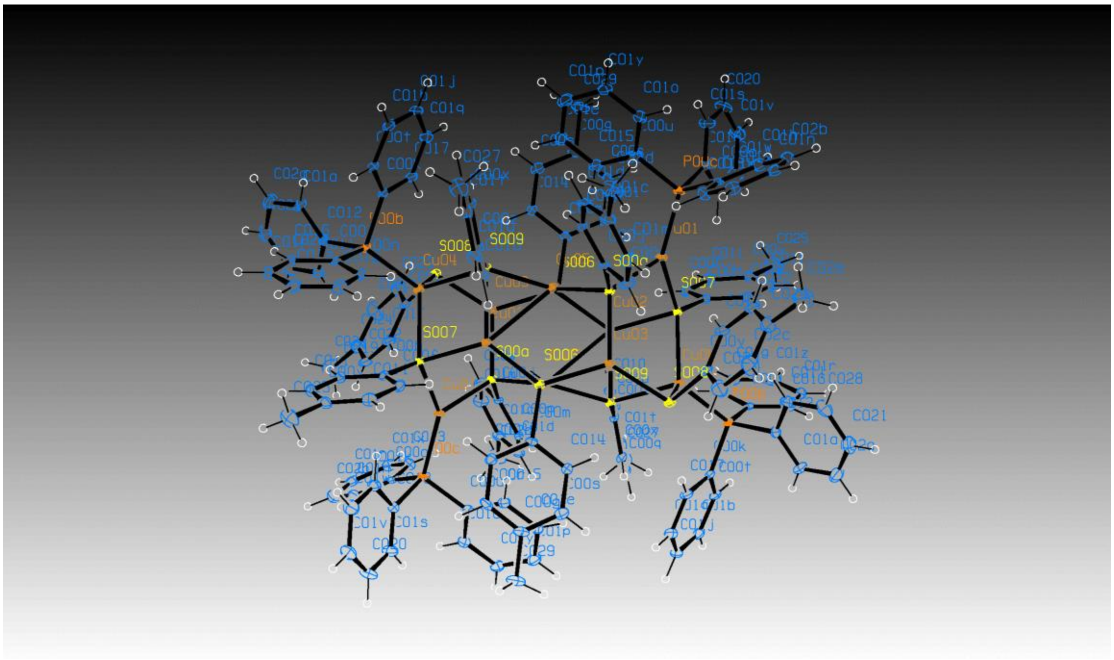

Supplementary Fig. 3. Representative Ortep diagram of Cu_{10}.[1] Thermal ellipsoids are set at 30% probability level. The CCDC number is 2174162.

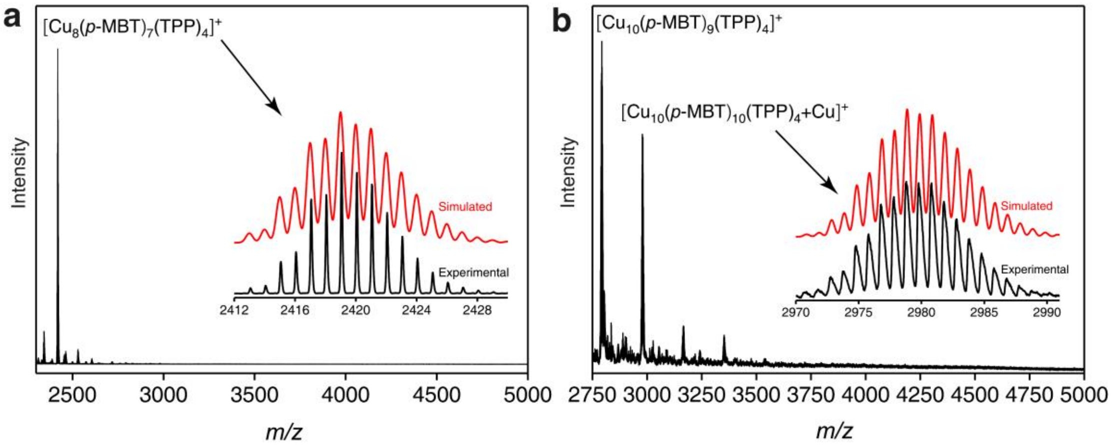

Supplementary Fig. 4. ESI-MS results of (**a**) Cu_8 and (**b**) Cu_{10} in the positive mode (the red line represents the simulated peak, and the black line represents the experimental peak).

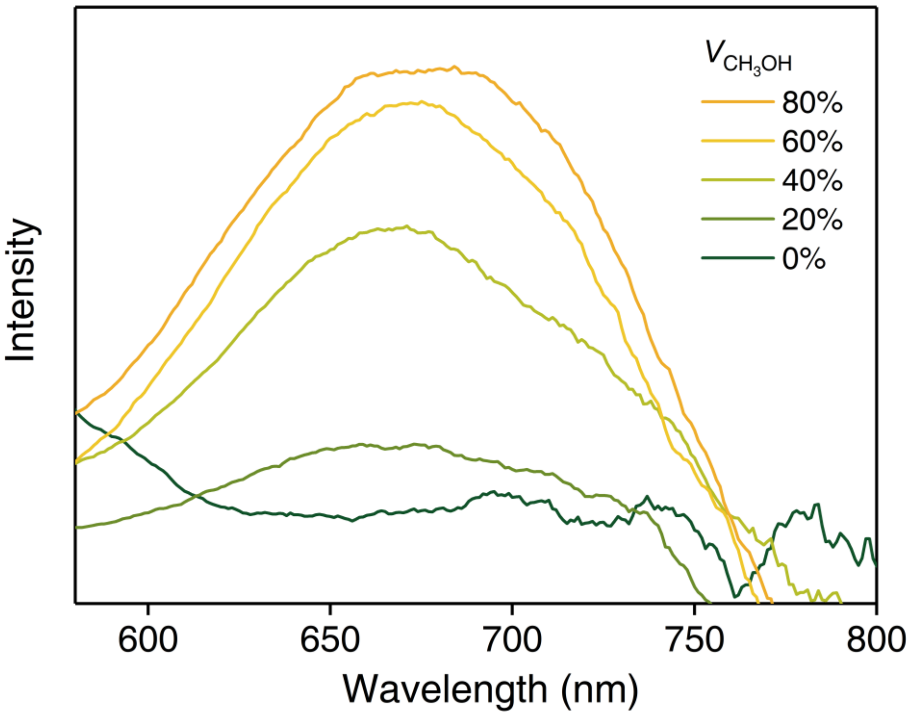

Supplementary Fig. 5. AIE performance of the Cu_{10} nanocluster in a mixed solution of CH_3OH/CH_2Cl_2 with different volume ratios (V_{CH_3OH}). The detailed AIE performance of the Cu_8 nanocluster refers to Supplementary Reference 2.

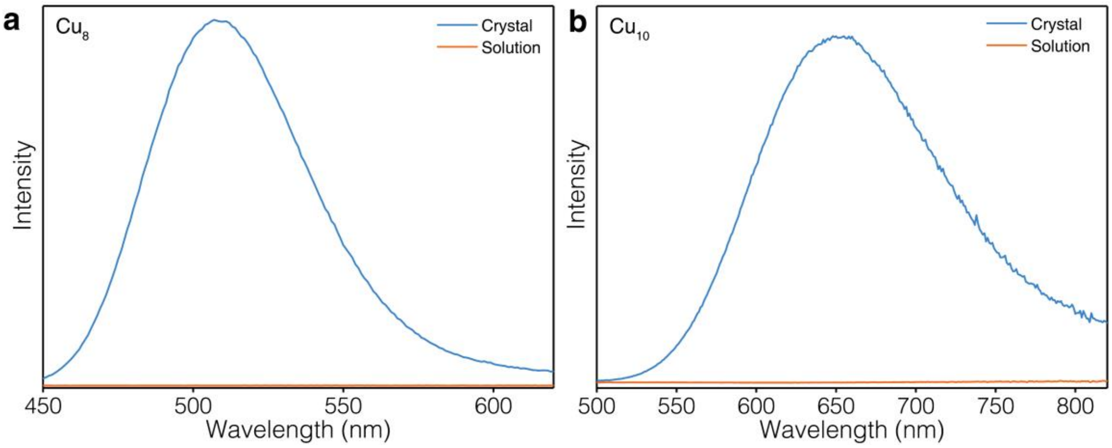

Supplementary Fig. 6. PL spectra. The PL spectra of (**a**) Cu_8 and (**b**) Cu_{10} nanocluster in crystal (blue) and solution (red) states.

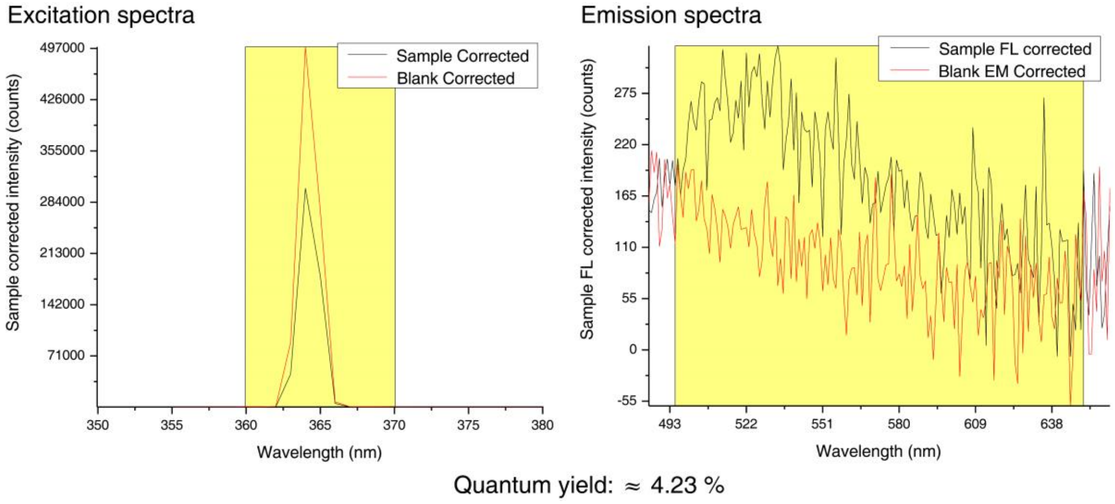

Supplementary Fig. 7. The absolute PL QY result of the Cu_8 nanocluster crystals at room temperature.

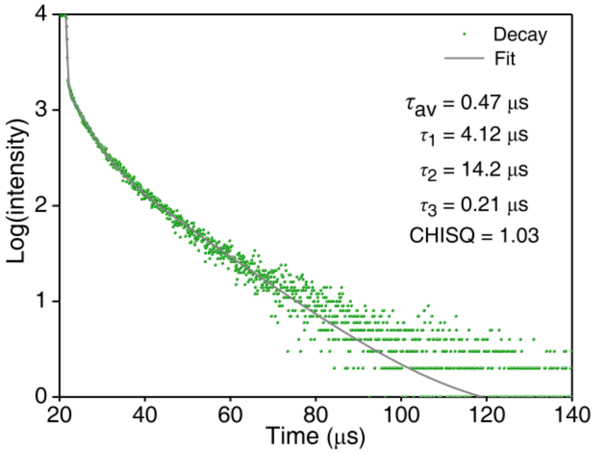

Supplementary Fig. 8. The emission lifetime of the Cu_8 nanocluster at room temperature.

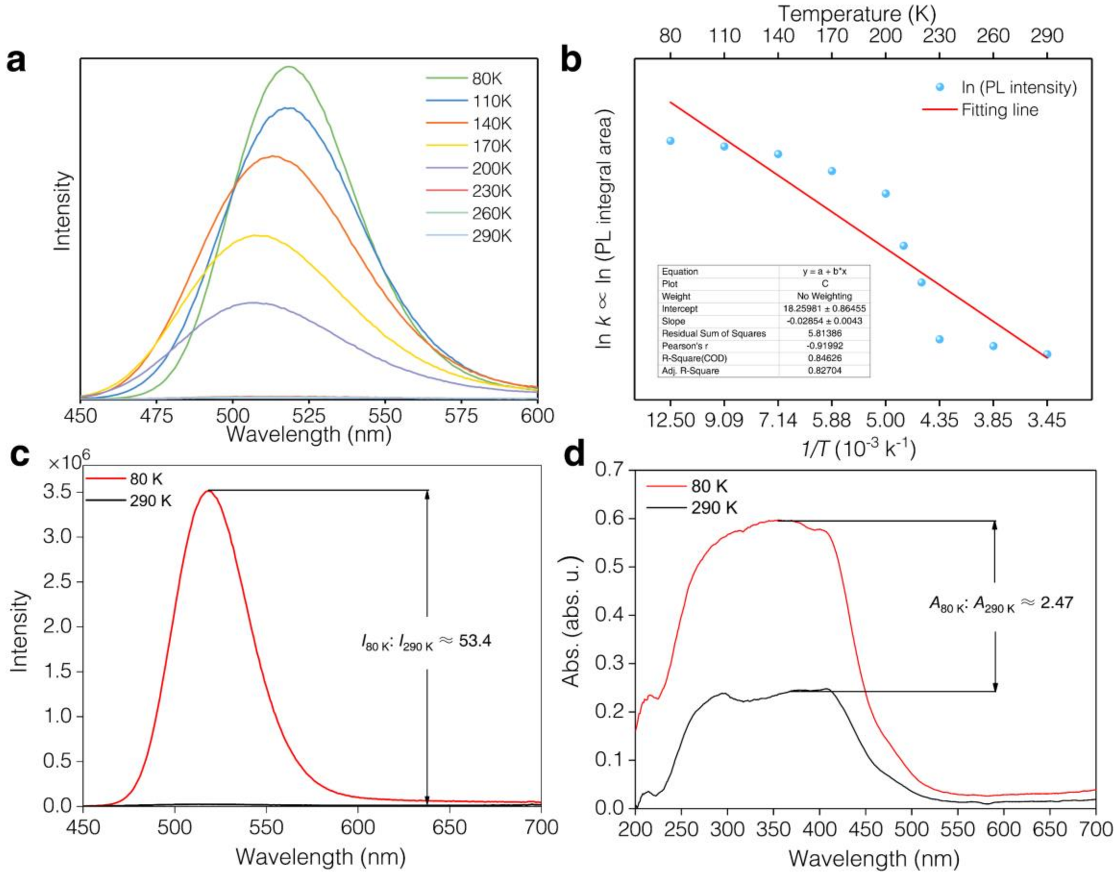

Supplementary Fig. 9. (**a**) Temperature-dependent PL spectra of the Cu_8 nanocluster. (**b**) The relationship between the *ln k* and *1/T*. The comparison of (**c**) integrated fluorescence intensity and (**d**) absorption intensity of the Cu_8 nanocluster at 290 K and 80 K, respectively.

According to the *Arrhenius Law*:

$$\ln k = -\frac{E_{\mathrm{a}}}{\mathrm{R}T} + \ln A \tag{1}$$

where k is the radiative rate, which is in direct proportion to the PL intensity. The relationship between the ln k and $1/T$ is given in Suppl. Fig. 7c. The results indicated that it might be the Arrhenius-type of behavior in some temperature intervals such as 80 K to 200 K, 200 K to 230 K, and 230 K to 290 K.

We tried to estimate the relative quantum yield at 80 K of Cu_8 nanocluster by using the integral area of the PL spectra and the absorption spectra intensity at different temperatures with the following equation:

$$Q_{80\,\mathrm{K}} = Q_{290\,\mathrm{K}} \frac{I_{80\,\mathrm{K}}}{I_{290\,\mathrm{K}}} \frac{A_{290\,\mathrm{K}}}{A_{80\,\mathrm{K}}} \tag{2}$$

where $Q_{80\,\mathrm{K}}$ is the quantum yield at 80 K, $Q_{290\,\mathrm{K}}$ is the quantum yield at 290 K, $I_{80\,\mathrm{K}}$ and $I_{290\,\mathrm{K}}$ are the integrated fluorescence intensity at 80 K and 290 K, respectively, and $A_{80\,\mathrm{K}}$ and $A_{290\,\mathrm{K}}$ are the absorption spectra intensity at 80 K and 290 K, respectively. Thus, the relative quantum yield of the Cu_8 nanocluster at 80 K was given as 88.5 %, not exceeding 100%.

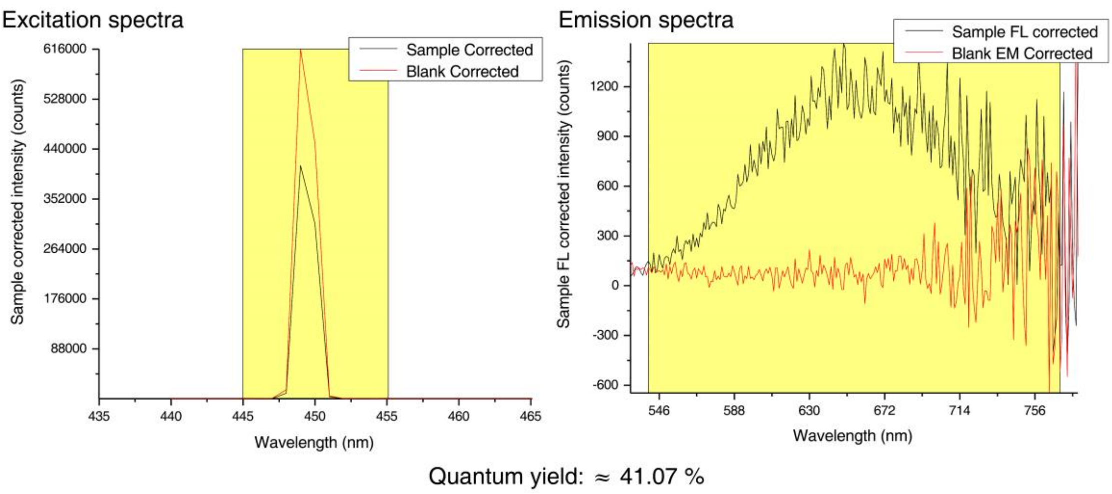

Supplementary Fig. 10. The absolute PL QY result of the Cu_{10} nanocluster crystals at room temperature.

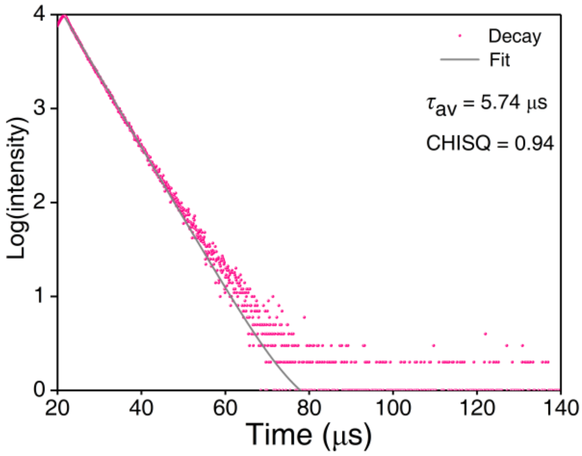

Supplementary Fig. 11. The emission lifetime of the Cu_{10} nanocluster at room temperature.

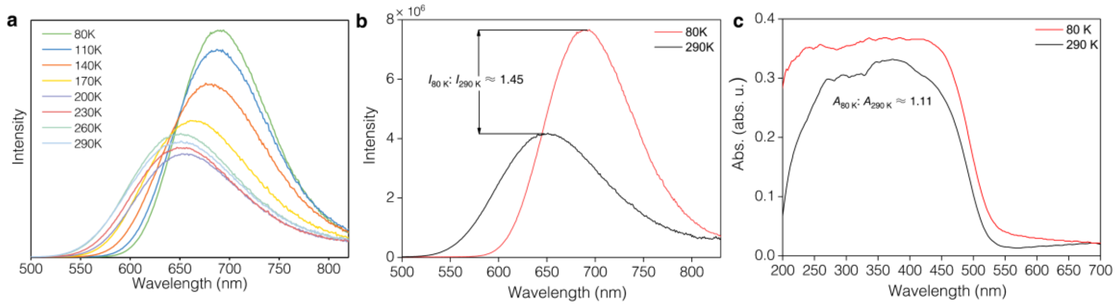

Supplementary Fig. 12. (**a**) Temperature-dependent PL spectra of Cu_{10} nanocluster. The comparison of (**b**) integrated fluorescence intensity and (**c**) absorption intensity of the Cu_{10} nanocluster at 290 K and 80 K, respectively. The relative quantum yield of the Cu_{10} nanocluster at 80 K was given as 53.5 %.

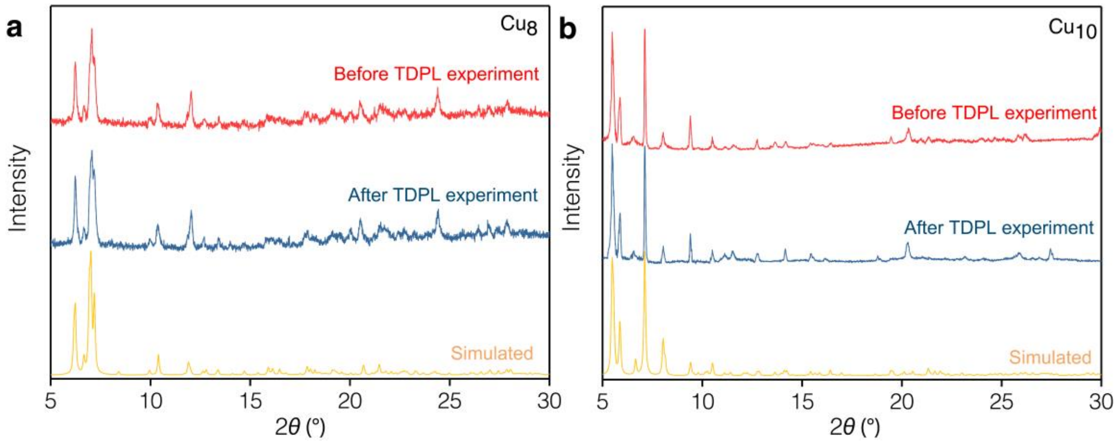

Supplementary Fig. 13. Comparison of the PXRD patterns of (**a**) Cu_8 and (**b**) Cu_{10} nanoclusters before and after the temperature-dependent PL (TDPL) test.

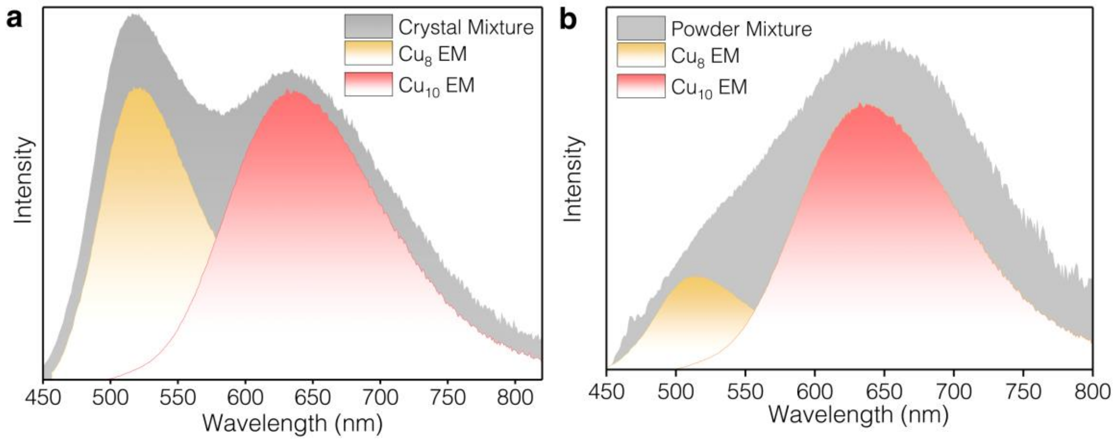

Supplementary Fig. 14. Physical mixture samples of (**a**) crystals and (**b**) amorphous powder of Cu_8 and Cu_{10} clusters.

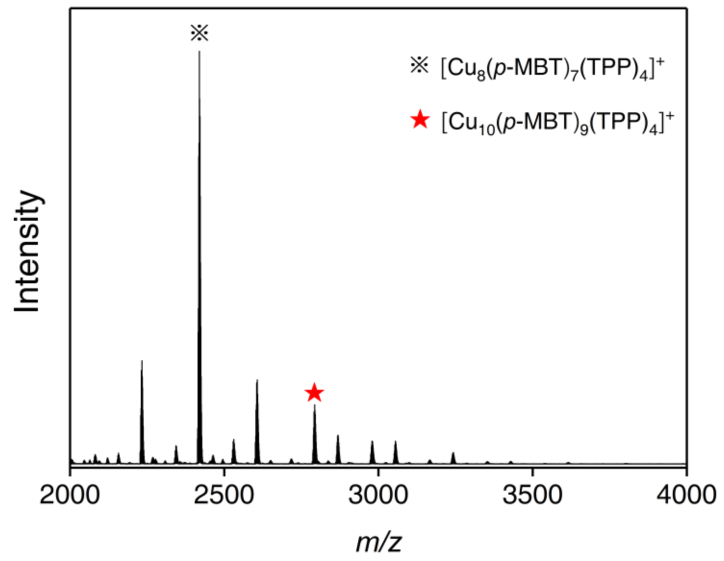

Supplementary Fig. 15. ESI-MS result of Cu_8 and Cu_{10} in the positive mode (the mother liquor before crystallization).

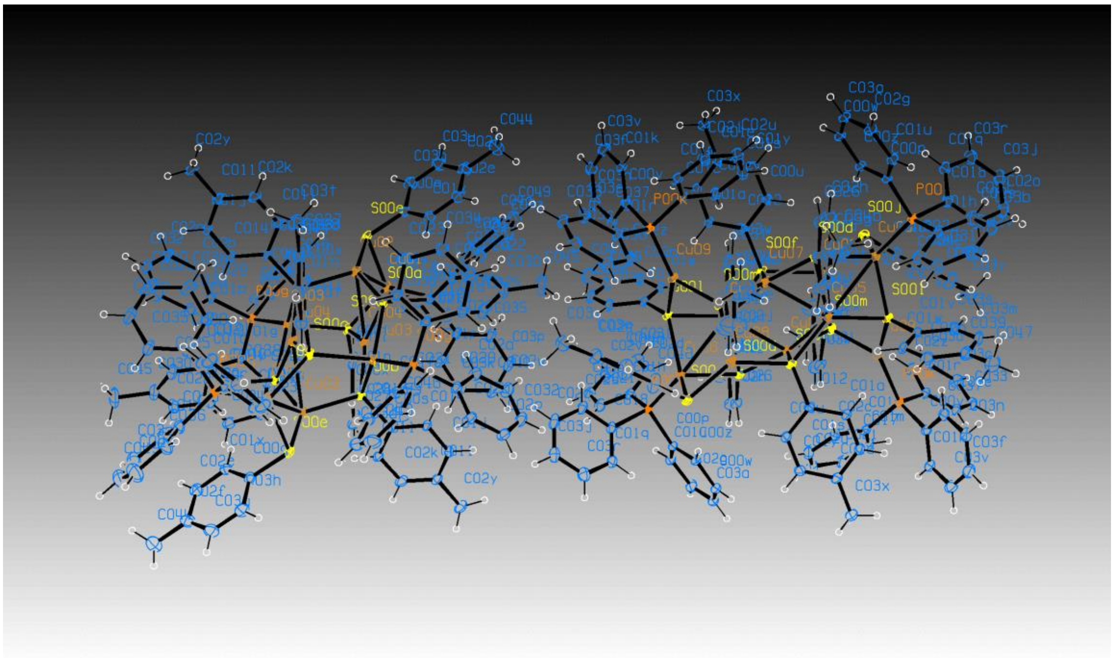

Supplementary Fig. 16. Representative Ortep diagram of $Cu_8@Cu_{10}$.[1] Thermal ellipsoids are set at 30% probability level. The CCDC number is 2174161.

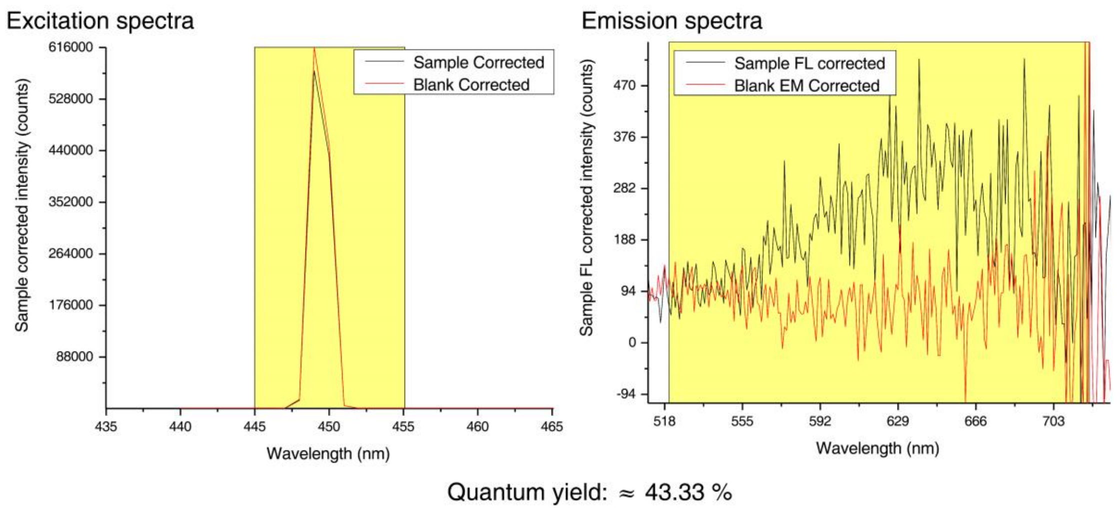

Supplementary Fig. 17. The absolute PL QY result of the Cu_8@Cu_{10} nanocluster crystals at room temperature.

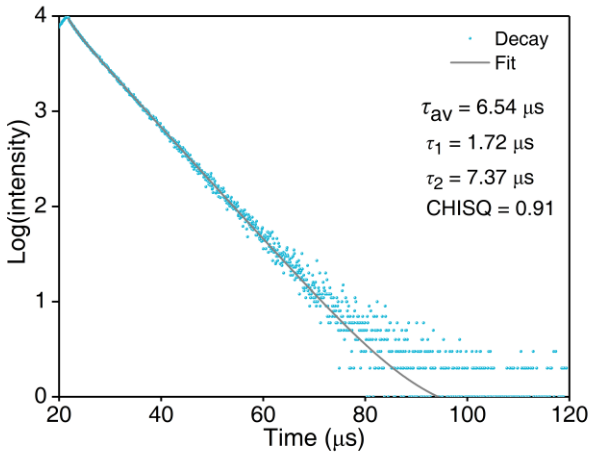

Supplementary Fig. 18. The emission lifetime of the Cu_8@Cu_{10} cocrystallized system at room temperature.

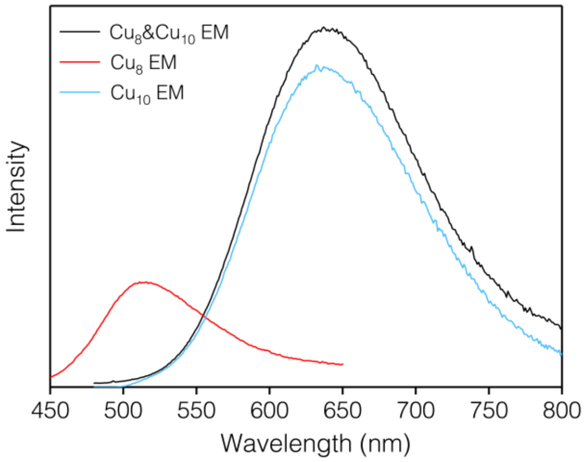

Supplementary Fig. 19. Comparison of the PL spectra of Cu_8, Cu_{10}, and Cu_8&Cu_{10} in crystal states at room temperature.

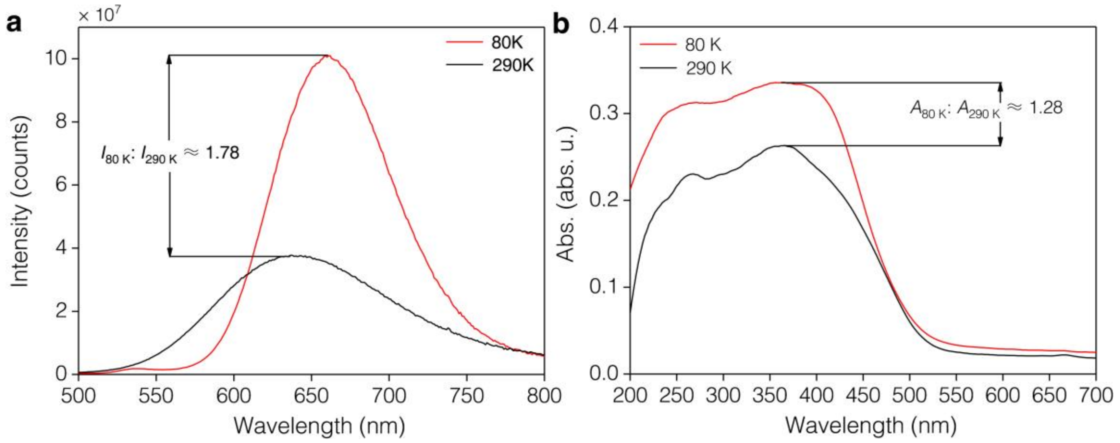

Supplementary Fig. 20. The comparison of (**a**) integrated fluorescence intensity and (**b**) absorption intensity of the Cu_8@Cu_{10} co-crystal at 290 K and 80 K, respectively. The relative quantum yield of the Cu_8@Cu_{10} co-crystal at 80 K was given as 60.4 %.

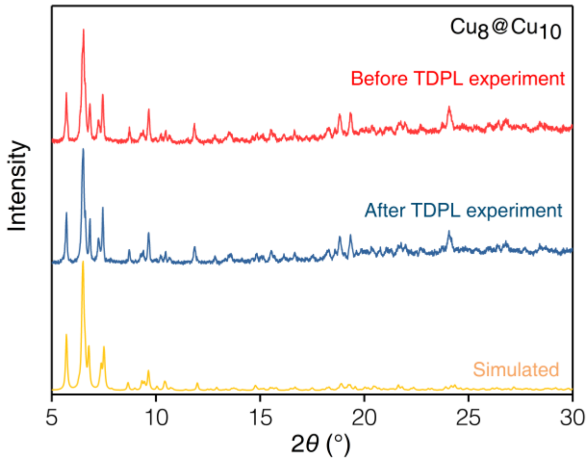

Supplementary Fig. 21. Comparison of the PXRD patterns of the cocrystallization Cu_8@Cu_{10} before and after the TDPL test.

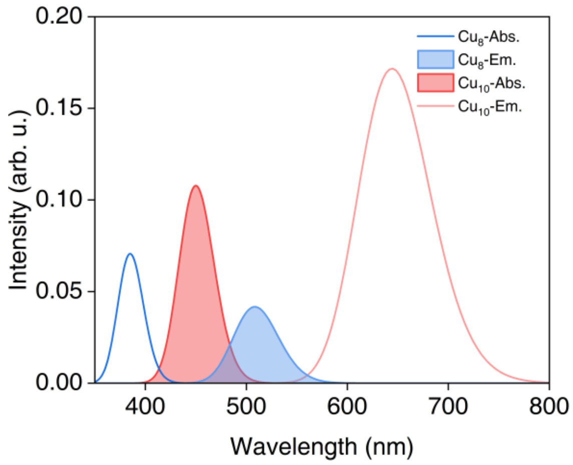

Supplementary Fig. 22. Summary of the experimental spectra of Cu_8 and Cu_{10} nanoclusters: uniform broadened spectra with a narrow broadening of 0.125 eV.

		Cu_8	Cu_{10}
Absorption	e^-	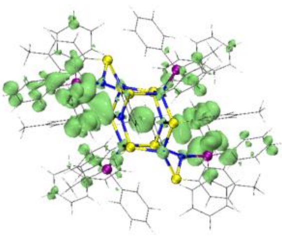	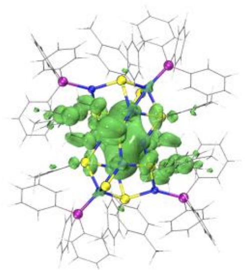
	h^+	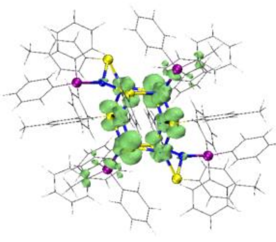	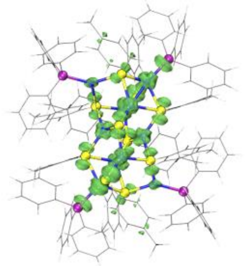
Emission	e^-	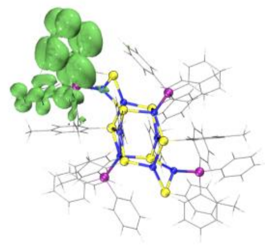	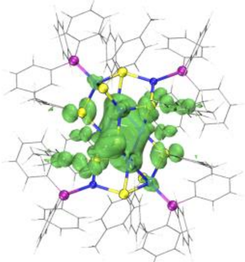
	h^+	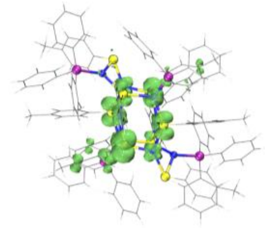	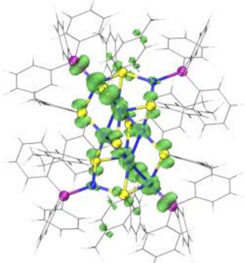

Supplementary Fig. 23. Spatial distribution of electrons and holes for absorptions and emissions of Cu_8 and Cu_{10} nanoclusters, respectively. Green surfaces are the electron/hole isosurfaces at 0.02 a.u.

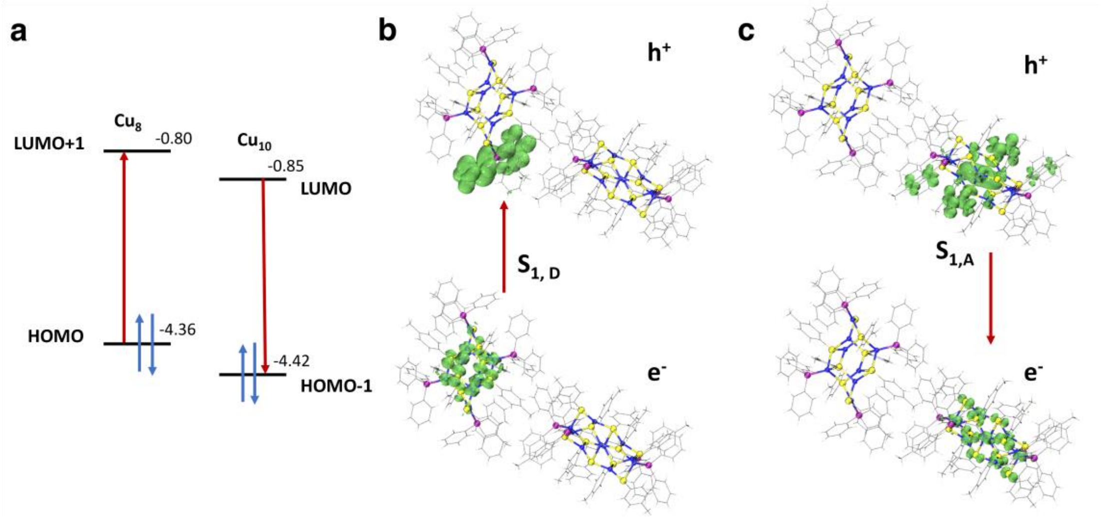

Supplementary Fig. 24. DFT calculated (**a**) relative energies of frontier orbitals in Cu_8@Cu_{10} cocrystal with the energy levels in units of eV and the spatial distribution of electrons/holes for (**b**) $S_{1,D}$ on Cu_8 and (**c**) $S_{1,A}$ on Cu_{10} with isosurfaces at 0.02 a.u.

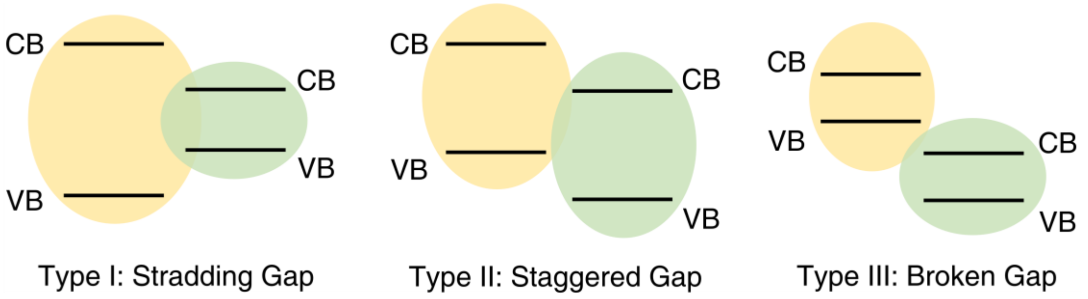

Supplementary Fig. 25. Three types of semiconductor heterojunctions organized by band alignment. CB: conduction band; VB: valence band.

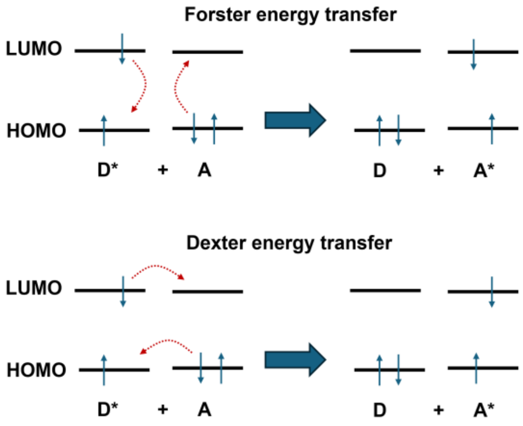

Supplementary Fig. 26. Schematic process of Förster resonance energy transfer and Dexter energy transfer.

LUMO	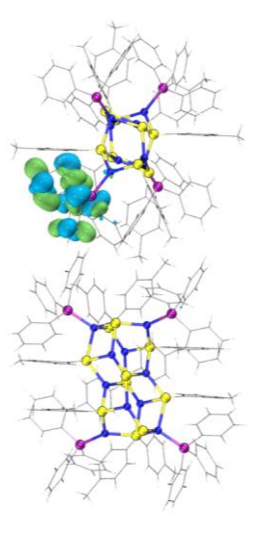	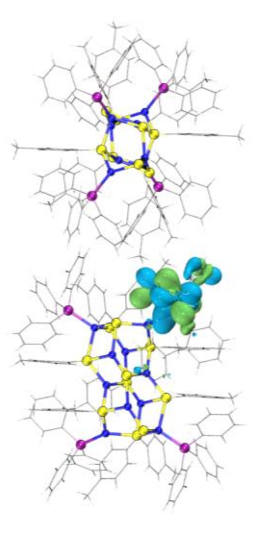
HOMO	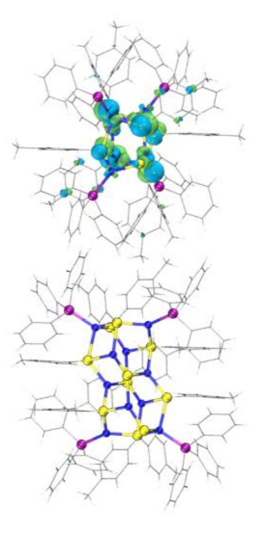	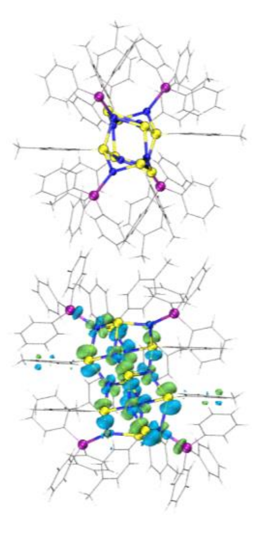
	Donor	Acceptor

Supplementary Fig. 27. Spatial distribution of HOMO and LUMO on donor (Cu_8) and acceptor (Cu_{10}).

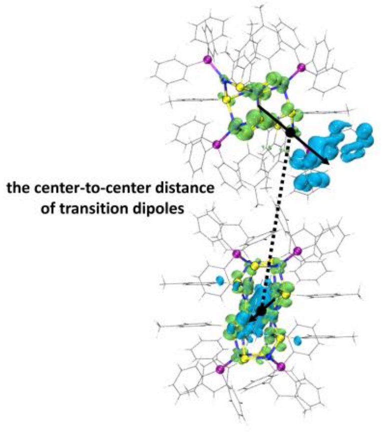

Supplementary Fig. 28. Schematic diagram for defining the center-to-center distance of transition dipoles of metal nanoclusters. The arrows label the transition dipoles from the center of holes to the center of electrons. The dashed line marks the center-to-center distance of two transition dipoles.

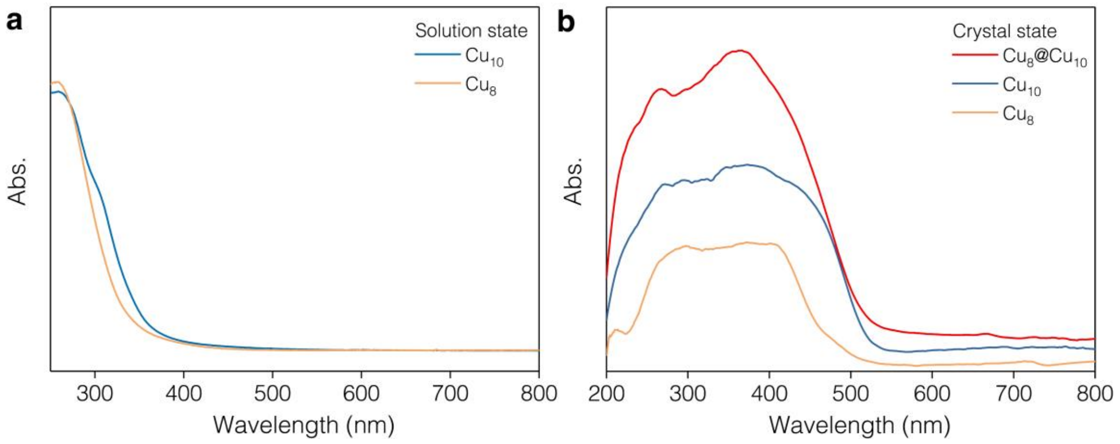

Supplementary Fig. 29. UV-vis spectra of the Cu nanoclusters in (**a**) solution and (**b**) crystal states.

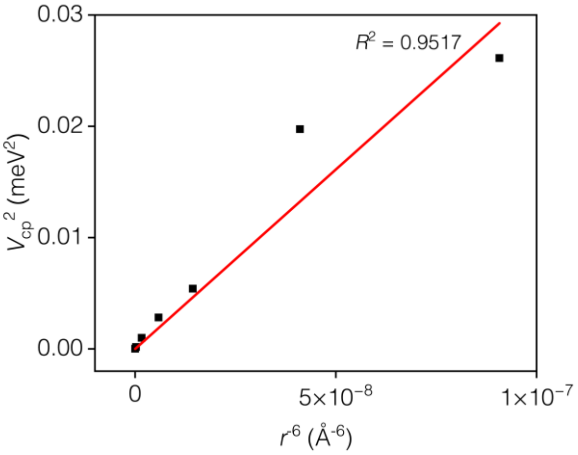

Supplementary Fig. 30. Square of electronic coupling strength ($V_{cp}{}^2$) with respect to -6 power of the distance (r^{-6}).

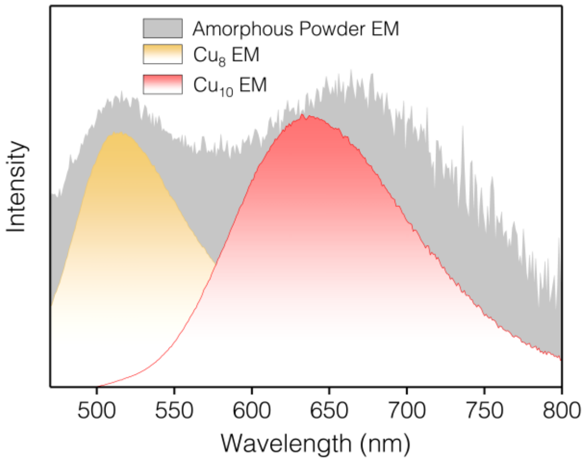

Supplementary Fig. 31. The PL spectra of amorphous powder after the solvent volatilization of cocrystalline $Cu_8@Cu_{10}$ clusters at room temperature.

Supplementary Table 1. Crystallographic data and structure refinement for Cu_8, Cu_{10}, and $Cu_8@Cu_{10}$ clusters.

Identification code	Cu_8	Cu_{10}	$Cu_8@Cu_{10}$
Empirical formula	$C_{128}H_{116}Cu_8P_4S_8$	$C_{142}H_{130}Cu_{10}P_4S_{10}$	$C_{270}H_{246}Cu_{18}P_8S_{18}$
Formula weight	2542.88	2916.33	5459.21
Temperature (K)	120	120	120
Crystal system	triclinic	triclinic	triclinic
Space group	$P\bar{1}$	$P\bar{1}$	$P\bar{1}$
a (Å)	14.6928(11)	13.4997(9)	14.4761(4)
b (Å)	15.6939(10)	16.9926(11)	17.7775(5)
c (Å)	15.9664(10)	17.6327(11)	25.9929(7)
α (°)	99.534(5)	114.461(5)	95.391(2)
β (°)	114.525(5)	107.878(5)	94.431(2)
γ (°)	110.688(5)	90.363(5)	111.039(2)
Volume ($Å^3$)	2915.0(4)	3462.2(4)	6171.2(3)
Z	1	1	1
ρ_{calc} (g cm^{-3})	1.449	1.399	1.469
μ (mm^{-1})	3.795	3.835	3.944
F(000)	1304.0	1492.0	2796.0
Crystal size (mm^3)	0.3 × 0.233 × 0.1	0.15 × 0.1 × 0.06	0.3 × 0.217 × 0.15
Radiation	Cu Kα (λ = 1.54186)	Cu Kα (λ = 1.54186)	Cu Kα (λ = 1.54186)
2Θ range for data collection (°)	6.972 to 124.998	6.964 to 124.994	6.748 to 124.986
Index ranges	-16≤h≤13, -11≤k≤18, -18≤l≤18	-15≤h≤14, -19≤k≤17, -8≤l≤20	-16≤h≤16, -20≤k≤20, -16≤l≤29
Reflections collected	19837	28739	51827
Independent reflections	9047 [R_{int} = 0.0291, R_{sigma} = 0.0315]	10837 [R_{int} = 0.0554, R_{sigma} = 0.0768]	19255 [R_{int} = 0.0632, R_{sigma} = 0.0688]
Data/restraints/parameters	9047/0/671	10837/0/753	19255/1620/1423
Goodness-of-fit on F^2	1.056	0.918	0.994
Final *R* indexes [I>=2σ (I)]	R_1 = 0.0481, wR_2 = 0.1328	R_1 = 0.0464, wR_2 = 0.1056	R_1 = 0.0609, wR_2 = 0.1612
Final *R* indexes [all data]	R_1 = 0.0542, wR_2 = 0.1375	R_1 = 0.0753, wR_2 = 0.1177	R_1 = 0.0924, wR_2 = 0.2177
Largest diff. peak/hole (e $Å^{-3}$)	1.19/-0.53	0.72/-0.42	1.74/-1.52

Supplementary Table 2: List of bond lengths in **Cu_8** nanocluster.

Bond type	Bond length range (Å)	Average length range (Å)
Cu-Cu	2.76-2.97	2.86
Cu-S	2.22-2.41	2.31
Cu-P	2.23-2.24	2.24

Supplementary Table 3: List of bond lengths in **Cu_{10}** nanocluster.

Bond type	Bond length range (Å)	Average length range (Å)
Cu-Cu	2.74-3.00	2.89
Cu-S	2.22-2.49	2.30
Cu-P	2.20-2.25	2.23

Supplementary Table 4. Photophysical Data of Cu_8, Cu_{10}, and Cu_8@Cu_{10} cluster.

**Cluster**	λ_{em}(nm)[a]	τ_{av} (μs) [b]	Φ_{em}[c]	κ_r (s^{-1}) [d]	κ_{nr} (s^{-1}) [e]
Cu_8	515	1.20	4.2 %	3.50×10^4	7.98×10^6
Cu_{10}	655	5.74	41.1 %	7.16×10^4	1.03×10^5
Cu_8@Cu_{10}	640	6.54	43.3 %	6.62×10^4	8.67×10^4

[a]Emission peak wavelength;

[b]Average emission lifetime determined;

[c]Emission quantum yield;

[d]Radiative rate constant calculated using the equation:

$$k_{\mathrm{r}} = \frac{\Phi_{\mathrm{em}}}{\tau_{\mathrm{av}}} \qquad (3)$$

[e]Non-radiative rate constant calculated using the equation:

$$k_{\mathrm{nr}} = \frac{1}{\tau_{\mathrm{av}}} - k_{\mathrm{r}} \qquad (4)$$

Supplementary Table 5. DFT calculated excitation energies (E), oscillator strength (f, a unitless quantity), centroid of holes/electrons, coupling strength (V_{cp}) between S_1 and S_2, FRET rate constant (k_{FRET}) ,and FRET efficiency (E_{FRET}) for the $Cu_8@Cu_{10}$ cocrystal.

**Distance (Å)**	14.9	17.0	20.3	23.5	29.1	39.8	65.0
**E_{S1} (eV)**	3.02	3.02	3.02	3.02	3.02	3.02	3.02
**f_{S1}**	0.016	0.016	0.016	0.016	0.016	0.016	0.016
**E_{S2} (eV)**	3.13	3.14	3.15	3.15	3.15	3.15	3.15
**f_{S2}**	0.007	0.007	0.008	0.008	0.009	0.009	0.009
**S_1 centroid of holes**	(8.04, -0.03, 0.03)	(7.46, 6.93, 9.78)	(8.66, 8.01, 7.29)	(9.78, 9.05, 4.68)	(11.74, 10.92, 0.48)	(15.55, 14.56, -8.09)	(25.16, 23.63, 29.07)
**S_1 centroid of electrons**	(7.74, -0.04, -0.03)	(7.40, 6.87, 9.98)	(8.61, 7.99, 7.37)	(9.75, 9.08, 4.83)	(11.67, 10.89, 0.57)	(15.55, 14.53, -7.90)	(25.17, 23.61, 29.10)
**S_2 centroid of holes**	(-8.68, 0.17, -0.18)	(0.09, 0.29, 25.66)	(0.03, 0.28, 25.77)	(-0.01, 0.28, 25.81)	(-0.03, 0.16, 25.84)	(-0.02, 0.01, 25.99)	(0.02, 0.04, 26.13)
**S_2 centroid of electrons**	(-4.93, 4.69, -1.24)	(-0.32, 5.49, 23.55)	(-0.27, 4.88, 23.82)	(-0.20, 4.05, 24.17)	(-0.12, 2.58, 24.84)	(-0.05, 0.92, 25.60)	(0.00, 0.15, 25.93)
**V_{cp} (meV)**	0.162	0.140	0.074	0.053	0.031	0.013	0.003
**k_{FRET} (s^{-1})**	7.59×10^7	5.73×10^7	1.57×10^7	8.20×10^6	2.88×10^6	4.78×10^5	2.43×10^4
**E_{FRET}***	97.7%	95.1%	87.1%	73.7%	43.7%	10.6%	0.6%

*
$$E_{\mathrm{FRET}} = \frac{1}{1+(r/R_0)^6} \tag{5}$$

Supplementary Table 6. electric transition dipole moments and its oscillator strength (a unitless quantity) among S_0, $S_{1,A}$, and $S_{2,D}$ for $Cu_8@Cu_{10}$ cluster.

		Transition dipole (arb. u.)				
Initial state	Final state	*X*	*Y*	*Z*	Energy difference (eV)	Oscillator strength
S0	$S_{1,A}$	0.3835	-0.6109	0.0030	3.0158	1.61×10^{-2}
S0	$S_{1,D}$	0.3500	0.0938	-0.0849	3.1327	6.90×10^{-3}
$S_{1,A}$	$S_{1,D}$	-0.0992	-0.0138	0.0009	0.1169	3.00×10^{-5}

Supplementary Note 1. The detailed PL lifetime report of Cu_8 nanocluster.

```
Calculated using 3 exponentials

Prompt data : (none)
Decay data : Cu8

The initial parameters are:

 Shift Value = Fixed @ 0       ch;        0            sec

 T1 Estimate = 60.96809 ch;       5.080685E-06   sec
 T2 Estimate = 121.9362 ch;       1.016137E-05   sec
 T3 Estimate = 243.8724 ch;       2.032274E-05   sec

 A Free
 B1 Free
 B2 Free
 B3 Free

 Prompt and decay LO = 258    ch; 2.150004E-05   sec
 Prompt and decay HI = 3854   ch; 3.211673E-04   sec

 Background on prompt = 0
 Time calibration = 8.33335E-08 sec/ch

The fitted parameters are:

 SHIFT = 0 ch

 T1    = 49.50233     ch; 4.125202E-06  sec    S.Dev = 7.659489E-08  sec
 T2    = 171.2956     ch; 1.427467E-05  sec    S.Dev = 9.899168E-08  sec
 T3    = 2.528087     ch; 2.106743E-07  sec    S.Dev = 2.080855E-09  sec
 A     = 0.5798025                  S.Dev = 2.058464E-02
 B1    = 1298.641     [ 40.09 Rel.Ampl][ 0.12 Alpha] S.Dev = 9.198021
 B2    = 422.6068     [ 45.14 Rel.Ampl][ 0.04 Alpha] S.Dev = 2.529208
 B3    = 9369.249     [ 14.77 Rel.Ampl][ 0.84 Alpha] S.Dev = 68.82784
 Average Life Time = 1.204958E-06  sec
 CHISQ = 1.036911        [ 3590 degrees of freedom ]

 Chi-squared Probability =   6.022932 percent
 Durbin-Watson Parameter =    1.50844
 Negative residuals     =    44.1201 percent
 Residuals < 1 s.dev    =   79.53851 percent
 Residuals < 2 s.dev    =   97.97053 percent
 Residuals < 3 s.dev    =   99.49958 percent
 Residuals < 4 s.dev    =    99.7776 percent
```

Supplementary Note 2. The detailed PL lifetime report of Cu_{10} nanocluster.

```
Calculated using 1 exponential

Prompt data : (none)
Decay data : Cu10

The initial parameters are:

 Shift Value = Fixed @ 0       ch;        0             sec

 T1 Estimate = 67.51666 ch;       5.6264E-06    sec

 A Free
 B1 Free

 Prompt and decay LO = 262    ch; 2.183338E-05   sec
 Prompt and decay HI = 2144   ch; 1.78667E-04    sec

 Background on prompt = 0
 Time calibration = 8.33335E-08 sec/ch

The fitted parameters are:

 SHIFT = 0 ch

 T1    = 68.88078     ch; 5.740076E-06  sec    S.Dev = 7.44892E-09   sec
 A     = 0.4720359                S.Dev = 2.877169E-02
 B1    = 9209.929     [ 100.00 Rel.Ampl][ 1.00 Alpha] S.Dev = 11.52668
 Average Life Time = 5.740076E-06  sec
 CHISQ = 0.9399104       [ 1880 degrees of freedom ]

 Chi-squared Probability =   96.92329 percent
 Durbin-Watson Parameter =   1.069499
 Negative residuals     =   50.13277 percent
 Residuals < 1 s.dev    =   74.72119 percent
 Residuals < 2 s.dev    =   94.90176 percent
 Residuals < 3 s.dev    =   99.25651 percent
 Residuals < 4 s.dev    =   99.68136 percent
```

Supplementary Note 3. The detailed PL lifetime report of $Cu_8@Cu_{10}$ nanocluster.

```
Calculated using 2 exponentials

Prompt data : (none)
Decay data : Cu8@Cu10

The initial parameters are:

 Shift Value = Fixed @ 0       ch;        0            sec

 T1 Estimate = 43.15645 ch;      3.596378E-06   sec
 T2 Estimate = 172.6258 ch;      1.438551E-05   sec

 A Free
 B1 Free
 B2 Free

 Prompt and decay LO = 262   ch; 2.183338E-05   sec
 Prompt and decay HI = 1505   ch; 1.254169E-04   sec

 Background on prompt = 0
 Time calibration = 8.33335E-08 sec/ch

The fitted parameters are:

 SHIFT = 0 ch

 T1    = 20.58366     ch; 1.715309E-06  sec    S.Dev = 1.091736E-07  sec
 T2    = 88.48396     ch; 7.373679E-06  sec    S.Dev = 1.139281E-08  sec
 A     = 0.5690722               S.Dev = 4.756662E-02
 B1    = 1383.29      [ 3.87 Rel.Ampl][ 0.15 Alpha] S.Dev = 35.01542
 B2    = 7983.066     [ 96.13 Rel.Ampl][ 0.85 Alpha] S.Dev = 12.46751
 Average Life Time = 6.53801E-06   sec
 CHISQ = 0.9065648       [ 1239 degrees of freedom ]

 Chi-squared Probability =    99.1523 percent
 Durbin-Watson Parameter =   1.902778
 Negative residuals     =    42.6045 percent
 Residuals < 1 s.dev    =   72.82958 percent
 Residuals < 2 s.dev    =   96.38264 percent
 Residuals < 3 s.dev    =   99.51768 percent
 Residuals < 4 s.dev    =       100 percent
```

Supplementary References

1. Farrugia, L. J. *WinGX* and *ORTEP for Windows*: an update. *J. Appl. Cryst.* **45**, 849-854 (2012).
2. Sun, P.-P. *et al.* Real-time fluorescent monitoring of kinetically controlled supramolecular self-assembly of atom-precise Cu_8 nanocluster. *Angew. Chem. Int. Ed.* **61**, e202200180 (2022).
