# Supplementary material for: Fluorescence resonance energy transfer in atomically precise metal nanoclusters by cocrystallization-induced spatial confinement
Source: Nat Commun. 2024 Jun 24;15:5351. doi: 10.1038/s41467-024-49735-7 (PMC11196639; doi:10.1038/s41467-024-49735-7)
Supplement: Supplementary file 3 — Description of Additional Supplementary Files [file 41467_2024_49735_MOESM3_ESM.pdf]

Description of Additional Supplementary Files

File Name: Supplementary Data 1

Description:

1. Cartesian coordinates of Cu at its S0 min.
2. Cartesian coordinates of Cu at its S1 min.
3. Cartesian coordinates of Cu at its S0 min.
4. Cartesian coordinates of Cu at its S1 min.
5. Cartesian coordinates of Cu8@Cu10 at its S0 min.
