# Supplementary material for: Fluorescence resonance energy transfer in atomically precise metal nanoclusters by cocrystallization-induced spatial confinement
Source: Nat Commun. 2024 Jun 24;15:5351. doi: 10.1038/s41467-024-49735-7 (PMC11196639; doi:10.1038/s41467-024-49735-7)

checkCIF/PLATON report

Structure factors have been supplied for datablock(s) 1

THIS REPORT IS FOR GUIDANCE ONLY. IF USED AS PART OF A REVIEW PROCEDURE FOR PUBLICATION, IT SHOULD NOT REPLACE THE EXPERTISE OF AN EXPERIENCED CRYSTALLOGRAPHIC REFEREE.

No syntax errors found. CIF dictionary Interpreting this report

Datablock: 1

Bond precision: C-C = 0.0066 A Wavelength=1.54186

Cell:	a=14.6928(11)	b=15.6939(10)	c=15.9664(10)
	alpha=99.534(5)	beta=114.525(5)	gamma=110.688(5)
Temperature:	120 K		

	Calculated	Reported
Volume	2915.0(4)	2915.0(4)
Space group	P -1	P -1
Hall group	-P 1	-P 1
Moiety formula	C128 H116 Cu8 P4 S8 [+ solvent]	C128 H116 Cu8 P4 S8
Sum formula	C128 H116 Cu8 P4 S8 [+ solvent]	C128 H116 Cu8 P4 S8
Mr	2542.98	2542.88
Dx,g cm-3	1.449	1.449
Z	1	1
Mu (mm-1)	3.795	3.795
F000	1304.0	1304.0
F000'	1294.28	
h,k,lmax	16,18,18	16,18,18
Nref	9292	9047
Tmin,Tmax	0.398,0.684	0.320,0.684
Tmin'	0.279	

Correction method= # Reported T Limits: Tmin=0.320 Tmax=0.684
AbsCorr = MULTI-SCAN

Data completeness= 0.974 Theta(max)= 62.499

R(reflections)= 0.0481(7776)   wR2(reflections)= 0.1375(9047)

S = 1.056   Npar= 671

The following ALERTS were generated. Each ALERT has the format
test-name_ALERT_alert-type_alert-level.
Click on the hyperlinks for more details of the test.

Alert level C

```
THETM01_ALERT_3_C  The value of sine(theta_max)/wavelength is less than 0.590
            Calculated sin(theta_max)/wavelength =    0.5753
PLAT029_ALERT_3_C _diffrn_measured_fraction_theta_full value Low .      0.974 Why?
PLAT094_ALERT_2_C Ratio of Maximum / Minimum Residual Density ....       2.24 Report
PLAT220_ALERT_2_C NonSolvent   Resd 1  C   Ueq(max)/Ueq(min) Range        3.1 Ratio
PLAT341_ALERT_3_C Low Bond Precision on  C-C Bonds ...............    0.00664 Ang.
PLAT911_ALERT_3_C Missing FCF Refl Between Thmin & STh/L=    0.575        244 Report
```

Alert level G

```
PLAT072_ALERT_2_G SHELXL First  Parameter in WGHT  Unusually Large       0.11 Report
PLAT154_ALERT_1_G The s.u.'s on the Cell Angles are Equal ..(Note)      0.005 Degree
PLAT232_ALERT_2_G Hirshfeld Test Diff (M-X)  Cu02     --S005_a   .        8.0 s.u.
PLAT232_ALERT_2_G Hirshfeld Test Diff (M-X)  Cu03     --S007     .        5.4 s.u.
PLAT232_ALERT_2_G Hirshfeld Test Diff (M-X)  Cu04     --S006     .        5.6 s.u.
PLAT605_ALERT_4_G Largest Solvent Accessible VOID in the Structure         91 A**3
PLAT720_ALERT_4_G Number of Unusual/Non-Standard Labels ..........        132 Note
PLAT793_ALERT_4_G Model has Chirality at S007        (Centro SPGR)          S Verify
PLAT794_ALERT_5_G Tentative Bond Valency for Cu01      (I)       .       1.00 Info
PLAT794_ALERT_5_G Tentative Bond Valency for Cu03      (I)       .       1.01 Info
PLAT794_ALERT_5_G Tentative Bond Valency for Cu04      (I)       .       1.12 Info
PLAT909_ALERT_3_G Percentage of I>2sig(I) Data at Theta(Max) Still        78% Note
PLAT910_ALERT_3_G Missing # of FCF Reflection(s) Below Theta(Min).          2 Note
PLAT933_ALERT_2_G Number of HKL-OMIT Records in Embedded .res File          6 Note
PLAT941_ALERT_3_G Average HKL Measurement Multiplicity ..........         2.2 Low
PLAT967_ALERT_5_G Note: Two-Theta Cutoff Value in Embedded .res ..      125.0 Degree
PLAT978_ALERT_2_G Number C-C Bonds with Positive Residual Density.          0 Info
```

0 **ALERT level A** = Most likely a serious problem - resolve or explain
0 **ALERT level B** = A potentially serious problem, consider carefully
6 **ALERT level C** = Check. Ensure it is not caused by an omission or oversight
17 **ALERT level G** = General information/check it is not something unexpected

1 ALERT type 1 CIF construction/syntax error, inconsistent or missing data
8 ALERT type 2 Indicator that the structure model may be wrong or deficient
7 ALERT type 3 Indicator that the structure quality may be low
3 ALERT type 4 Improvement, methodology, query or suggestion
4 ALERT type 5 Informative message, check

It is advisable to attempt to resolve as many as possible of the alerts in all categories. Often the minor alerts point to easily fixed oversights, errors and omissions in your CIF or refinement strategy, so attention to these fine details can be worthwhile. In order to resolve some of the more serious problems it may be necessary to carry out additional measurements or structure refinements. However, the purpose of your study may justify the reported deviations and the more serious of these should normally be commented upon in the discussion or experimental section of a paper or in the "special_details" fields of the CIF. checkCIF was carefully designed to identify outliers and unusual parameters, but every test has its limitations and alerts that are not important in a particular case may appear. Conversely, the absence of alerts does not guarantee there are no aspects of the results needing attention. It is up to the individual to critically assess their own results and, if necessary, seek expert advice.

Publication of your CIF in IUCr journals

A basic structural check has been run on your CIF. These basic checks will be run on all CIFs submitted for publication in IUCr journals (*Acta Crystallographica*, *Journal of Applied Crystallography*, *Journal of Synchrotron Radiation*); however, if you intend to submit to *Acta Crystallographica Section C* or *E* or *IUCrData*, you should make sure that full publication checks are run on the final version of your CIF prior to submission.

Publication of your CIF in other journals

Please refer to the *Notes for Authors* of the relevant journal for any special instructions relating to CIF submission.

PLATON version of 18/05/2022; check.def file version of 17/05/2022

Datablock 1 - ellipsoid plot

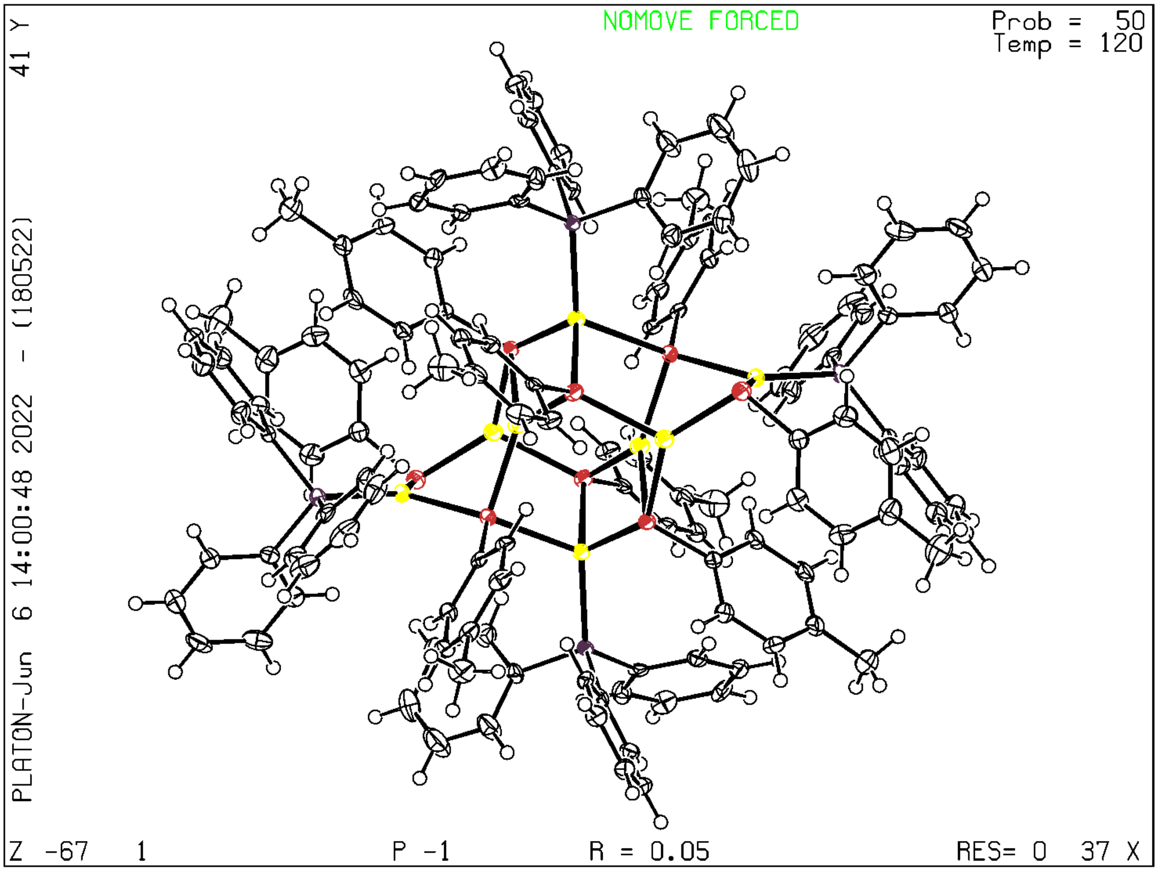

Supplement: Supplementary file 4 — Supplementary Data 1 [file 41467_2024_49735_MOESM4_ESM.zip › Suppl. Data/CIFs/checkcif for Cu8.pdf]
