# Supplementary material for: Fluorescence resonance energy transfer in atomically precise metal nanoclusters by cocrystallization-induced spatial confinement
Source: Nat Commun. 2024 Jun 24;15:5351. doi: 10.1038/s41467-024-49735-7 (PMC11196639; doi:10.1038/s41467-024-49735-7)

checkCIF/PLATON report

Structure factors have been supplied for datablock(s) 1

THIS REPORT IS FOR GUIDANCE ONLY. IF USED AS PART OF A REVIEW PROCEDURE FOR PUBLICATION, IT SHOULD NOT REPLACE THE EXPERTISE OF AN EXPERIENCED CRYSTALLOGRAPHIC REFEREE.

No syntax errors found. CIF dictionary Interpreting this report

Datablock: 1

```
Bond precision:   C-C = 0.0121 A          Wavelength=1.54186

Cell:           a=14.4761(4)      b=17.7775(5)      c=25.9929(7)
                alpha=95.391(2)   beta=94.431(2)    gamma=111.039(2)
Temperature:    120 K
```

	Calculated	Reported
Volume	6171.2(3)	6171.2(3)
Space group	P -1	P -1
Hall group	-P 1	-P 1
Moiety formula	C142 H130 Cu10 P4 S10, C128 H116 Cu8 P4 S8 [+ solvent]	2.109(C128 H116 Cu8 P4 S8)
Sum formula	C270 H246 Cu18 P8 S18 [+ solvent]	C270 H246 Cu18 P8 S18
Mr	5459.42	5459.21
Dx,g cm-3	1.469	1.469
Z	1	1
Mu (mm-1)	3.944	3.944
F000	2796.0	2796.0
F000’	2773.52	
h,k,lmax	16,20,29	17,21,31
Nref	19696	19255
Tmin,Tmax	0.412,0.553	0.100,0.720
Tmin’	0.266	

```
Correction method= # Reported T Limits: Tmin=0.100 Tmax=0.720
AbsCorr = MULTI-SCAN

Data completeness= 0.978            Theta(max)= 62.493
```

R(reflections)= 0.0609(13570) wR2(reflections)= 0.2177(19255)

S = 0.994 Npar= 1423

The following ALERTS were generated. Each ALERT has the format
test-name_ALERT_alert-type_alert-level.
Click on the hyperlinks for more details of the test.

Alert level C

```
REFLE01_ALERT_3_C  The _reflns_threshold_multiplier given is >= 4
            Premultiplier =    4.00
REFLE01_ALERT_3_C  The _reflns_threshold_multiplier given is >= 4
            Premultiplier =    4.00
THETM01_ALERT_3_C  The value of sine(theta_max)/wavelength is less than 0.590
            Calculated sin(theta_max)/wavelength =    0.5752
PLAT029_ALERT_3_C _diffrn_measured_fraction_theta_full value Low .      0.978 Why?
PLAT220_ALERT_2_C NonSolvent   Resd 1  C   Ueq(max)/Ueq(min) Range        4.1 Ratio
PLAT222_ALERT_3_C NonSolvent Resd 1  H   Uiso(max)/Uiso(min) Range        4.6 Ratio
PLAT241_ALERT_2_C High   'MainMol' Ueq as Compared to Neighbors of       C04F Check
PLAT334_ALERT_2_C Small Aver. Benzene C-C Dist C013     -C048            1.37 Ang.
PLAT341_ALERT_3_C Low Bond Precision on  C-C Bonds ...............    0.01207 Ang.
PLAT911_ALERT_3_C Missing FCF Refl Between Thmin & STh/L=    0.575        438 Report
PLAT918_ALERT_3_C Reflection(s) with I(obs) much Smaller I(calc) .         24 Check
PLAT977_ALERT_2_C Check Negative Difference Density on H00R            -0.44 eA-3
PLAT977_ALERT_2_C Check Negative Difference Density on H01S            -0.56 eA-3
PLAT977_ALERT_2_C Check Negative Difference Density on H025            -0.33 eA-3
PLAT977_ALERT_2_C Check Negative Difference Density on H038            -0.31 eA-3
PLAT977_ALERT_2_C Check Negative Difference Density on H03A            -0.39 eA-3
PLAT977_ALERT_2_C Check Negative Difference Density on H03             -0.34 eA-3
PLAT977_ALERT_2_C Check Negative Difference Density on H04R            -0.36 eA-3
PLAT977_ALERT_2_C Check Negative Difference Density on H04H            -0.47 eA-3
```

Alert level G

```
FORMU01_ALERT_1_G  There is a discrepancy between the atom counts in the
            _chemical_formula_sum and _chemical_formula_moiety. This is
            usually due to the moiety formula being in the wrong format.
            Atom count from _chemical_formula_sum:   C270 H246 Cu18 P8 S18
            Atom count from _chemical_formula_moiety:C269.9520 H244.6439 Cu16.872
PLAT003_ALERT_2_G Number of Uiso or Uij Restrained non-H Atoms ...        135 Report
PLAT042_ALERT_1_G Calc. and Reported Moiety Formula Strings Differ     Please Check
PLAT072_ALERT_2_G SHELXL First  Parameter in WGHT  Unusually Large       0.16 Report
PLAT154_ALERT_1_G The s.u.'s on the Cell Angles are Equal ..(Note)      0.002 Degree
PLAT178_ALERT_4_G The CIF-Embedded .res File Contains SIMU Records          1 Report
PLAT186_ALERT_4_G The CIF-Embedded .res File Contains ISOR Records          1 Report
PLAT605_ALERT_4_G Largest Solvent Accessible VOID in the Structure        185 A**3
PLAT720_ALERT_4_G Number of Unusual/Non-Standard Labels ..........        277 Note
PLAT793_ALERT_4_G Model has Chirality at S00B        (Centro SPGR)          R Verify
PLAT793_ALERT_4_G Model has Chirality at S00C        (Centro SPGR)          S Verify
PLAT794_ALERT_5_G Tentative Bond Valency for Cu01      (I)       .       1.00 Info
PLAT794_ALERT_5_G Tentative Bond Valency for Cu02      (I)       .       1.13 Info
PLAT794_ALERT_5_G Tentative Bond Valency for Cu04      (I)       .       0.99 Info
PLAT794_ALERT_5_G Tentative Bond Valency for Cu05      (I)       .       1.02 Info
PLAT794_ALERT_5_G Tentative Bond Valency for Cu07      (I)       .       1.11 Info
```

```
PLAT794_ALERT_5_G Tentative Bond Valency for Cu08      (I)       .       1.17 Info
PLAT794_ALERT_5_G Tentative Bond Valency for Cu09      (I)       .       1.09 Info
PLAT860_ALERT_3_G Number of Least-Squares Restraints ............        1620 Note
PLAT909_ALERT_3_G Percentage of I>2sig(I) Data at Theta(Max) Still        54% Note
PLAT910_ALERT_3_G Missing # of FCF Reflection(s) Below Theta(Min).          4 Note
PLAT933_ALERT_2_G Number of OMIT Records in Embedded .res File ...          1 Note
PLAT941_ALERT_3_G Average HKL Measurement Multiplicity ..........         2.7 Low
PLAT978_ALERT_2_G Number C-C Bonds with Positive Residual Density.          0 Info
```

```
   0 ALERT level A = Most likely a serious problem - resolve or explain
   0 ALERT level B = A potentially serious problem, consider carefully
  19 ALERT level C = Check. Ensure it is not caused by an omission or oversight
  24 ALERT level G = General information/check it is not something unexpected

   3 ALERT type 1 CIF construction/syntax error, inconsistent or missing data
  15 ALERT type 2 Indicator that the structure model may be wrong or deficient
  12 ALERT type 3 Indicator that the structure quality may be low
   6 ALERT type 4 Improvement, methodology, query or suggestion
   7 ALERT type 5 Informative message, check
```

It is advisable to attempt to resolve as many as possible of the alerts in all categories. Often the minor alerts point to easily fixed oversights, errors and omissions in your CIF or refinement strategy, so attention to these fine details can be worthwhile. In order to resolve some of the more serious problems it may be necessary to carry out additional measurements or structure refinements. However, the purpose of your study may justify the reported deviations and the more serious of these should normally be commented upon in the discussion or experimental section of a paper or in the "special_details" fields of the CIF. checkCIF was carefully designed to identify outliers and unusual parameters, but every test has its limitations and alerts that are not important in a particular case may appear. Conversely, the absence of alerts does not guarantee there are no aspects of the results needing attention. It is up to the individual to critically assess their own results and, if necessary, seek expert advice.

Publication of your CIF in IUCr journals

A basic structural check has been run on your CIF. These basic checks will be run on all CIFs submitted for publication in IUCr journals (*Acta Crystallographica*, *Journal of Applied Crystallography*, *Journal of Synchrotron Radiation*); however, if you intend to submit to *Acta Crystallographica Section C* or *E* or *IUCrData*, you should make sure that full publication checks are run on the final version of your CIF prior to submission.

Publication of your CIF in other journals

Please refer to the *Notes for Authors* of the relevant journal for any special instructions relating to CIF submission.

PLATON version of 13/07/2021; check.def file version of 13/07/2021

Datablock 1 - ellipsoid plot

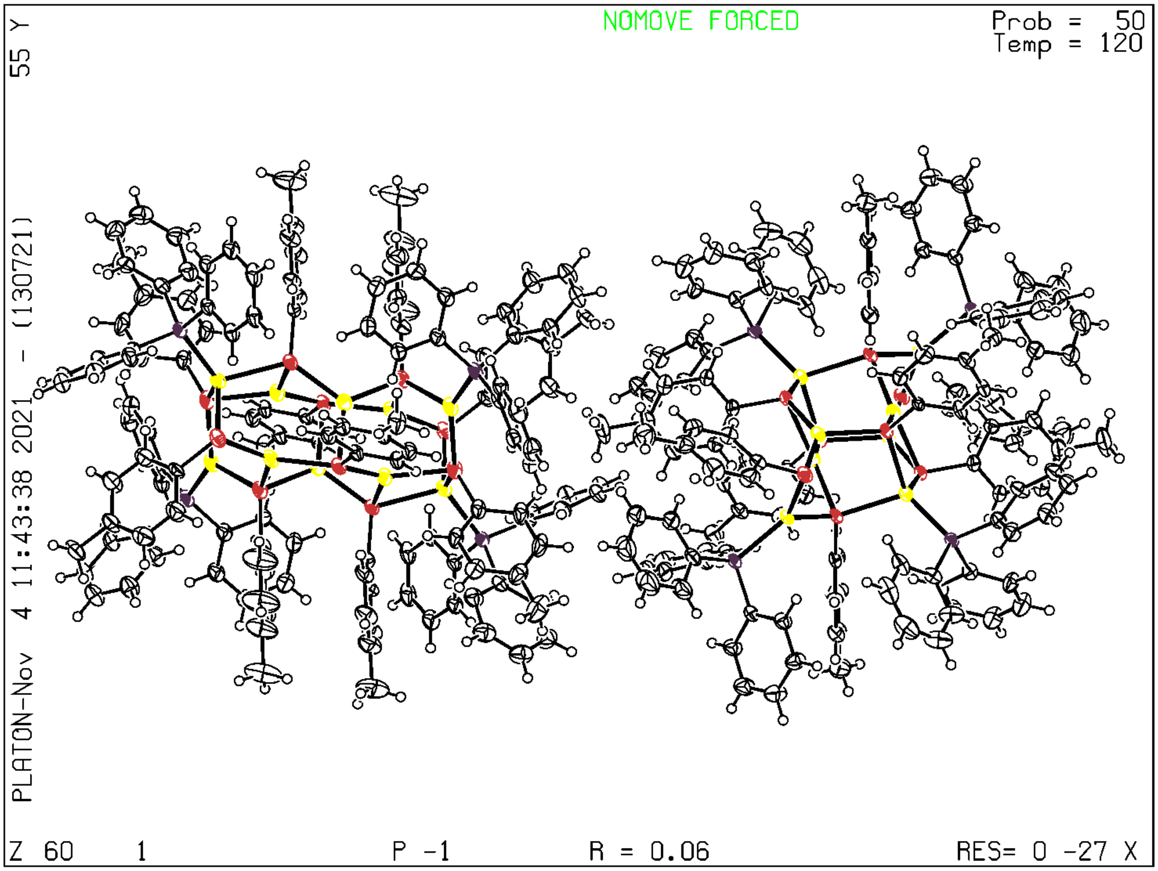

Supplement: Supplementary file 4 — Supplementary Data 1 [file 41467_2024_49735_MOESM4_ESM.zip › Suppl. Data/CIFs/checkcif for Cu8@Cu10.pdf]
